# Cytosolic DNA sensors in neurodegenerative diseases: from physiological defenders to pathological culprits

**DOI:** 10.1038/s44321-024-00046-w

**Published:** 2024-03-11

**Authors:** Jiatian Xie, Jinping Cheng, Ho Ko, Yamei Tang

**Affiliations:** 1 Department of Neurology, Sun Yat-Sen Memorial Hospital, Sun Yat-sen University, Guangzhou, 510120 China; 2 Brain Research Center, Sun Yat-sen Memorial Hospital, Sun Yat‑sen University, Guangzhou, 510120 China; 3 Nanhai Translational Innovation Center of Precision Immunology, Sun Yat-sen Memorial Hospital, Foshan, 528200 China; 4 Division of Neurology, Department of Medicine and Therapeutics & Li Ka Shing Institute of Health Sciences, Faculty of Medicine, The Chinese University of Hong Kong, Shatin, Hong Kong China

**Keywords:** Cytosolic DNA, Neurodegeneration, STING, Innate Immune, Type 1 Interferon, Immunology, Neuroscience

## Abstract

Cytosolic DNA sensors are a group of pattern recognition receptors (PRRs) that vary in structures, molecular mechanisms, and origins but share a common function to detect intracellular microbial DNA and trigger the innate immune response like type 1 interferon production and autophagy. Cytosolic DNA sensors have been proven as indispensable defenders against the invasion of many pathogens; however, growing evidence shows that self-DNA misplacement to cytoplasm also frequently occurs in non-infectious circumstances. Accumulation of cytosolic DNA causes improper activation of cytosolic DNA sensors and triggers an abnormal autoimmune response, that significantly promotes pathological progression. Neurodegenerative diseases are a group of neurological disorders characterized by neuron loss and still lack effective treatments due to a limited understanding of pathogenesis. But current research has found a solid relationship between neurodegenerative diseases and cytosolic DNA sensing pathways. This review summarizes profiles of several major cytosolic DNA sensors and their common adaptor protein STING. It also discusses both the beneficial and detrimental roles of cytosolic DNA sensors in the genesis and progression of neurodegenerative diseases.

## Abstract (web summary)

This review discusses the role of physiological roles of cytosolic DNA sensors and how they can become overactivated in the central nervous system and contribute to pathological progress of neurodegenerative diseases.

## Graphical abstract

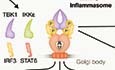

## Introduction

Neurodegenerative diseases are a group of lethal neurological disorders that are characterized by progressive neural function loss, including cognitive and motor function. As the global population age is rapidly increasing, neurodegenerative diseases have become one of the most important public health challenges in the world (Nichols et al, 2022). However, the pathogenesis of most types of neurodegenerative diseases remains unknown, and no effective treatments have been found.

Neurodegenerative diseases usually differ in pathological features, lesion regions, and primary symptoms, but all are characterized by continuous and irreversible neuron impairment and loss, implying there is underlying common pathogenesis. DNA damage and lateral cytosolic DNA leakage are universal phenomena in brain diseases (Brasnjevic et al, 2008). When neuronal damage and death occur, a large amount of self-DNA is released to extracellular space and absorbed by resident immune cells that are able to trigger an inflammatory response, therefore amplifying neuronal damage through a “cell death-inflammation” vicious loop (Erdal et al, 2017). Increasing oxidative stress also initiates DNA damage and leakage both in the nucleus and mitochondria and contributes to neuronal death (Coppedè and Migliore, 2015).

Soon after DNA is released in the cytoplasm, it activates cytosolic DNA sensors. Cytosolic DNA sensors are important components of the innate immune defense system. They originally serve as detectors of DNA from microbes such as viruses and intracellular bacteria, triggering immune responses to restrict microbial invasion. However, in the context of neurodegenerative diseases, these defenders behave with abnormal activation due to accumulation of cytosolic self-DNA, resulting in detrimental immune response and further neural damage. In this review, we discuss the major kinds of cytosolic DNA sensors, including their features, and how they become culprits in various types of neurodegenerative diseases.

## Main cytosolic DNA sensors: characters and roles

**Figure 1:**
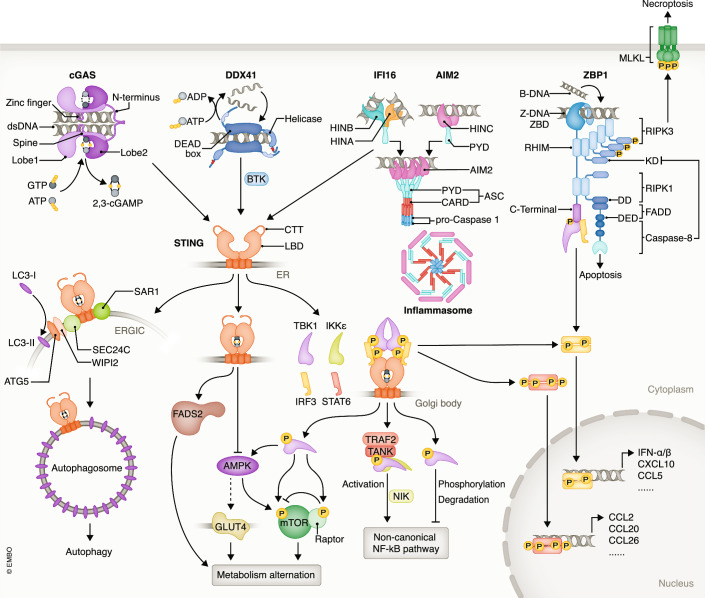
The main cytosolic DNA sensors and their downstream pathways. Functional dimeric cGAS catalyzes the conversion of ATP and GTP into 2,3-cGAMP when bound to dsDNA. DDX41 only binds one strand of DNA, and its helicase domain reserves the ability to unwind or anneal DNA strands. The HIN200 family members, IFI16 and AIM2, tend to oligomerize in order to detect dsDNA. In addition to detecting dsDNA, ZBP1 is capable of transforming Z-DNA to B-DNA. cGAS, DDX41, and IFI16 all activate STING to initiate their downstream effects, with cGAS relying on 2,3-cGAMP, while IFI16 and DDX41 require direct self-binding. STING mainly triggers the release of inflammatory cytokines such as IFN-I through the TBK1/IKKε-IRF3/STAT6 pathway and also contributes to autophagy and metabolic alterations. ZBP1 can activate the TBK1-IRF3 pathway independently of STING and induce necroptosis or apoptosis depending on the balance between RIPK1 and RIPK3. Unlike other cytosolic DNA sensors, AIM2 does not exert its biological effect through STING or IFN-I; instead, AIM2 takes part in the formation of an inflammasome. IFI16 also recruits inflammasomes, but the mechanism that determines whether IFI16 prefers to bind inflammasomes or STING remains unknown.

Cytosolic DNA sensors are the bridge between DNA damage, leakage, and neuron loss in neurodegenerative diseases. All able to detect and bind DNA in cytoplasm, these molecules can induce direct cell death and/or neuroinflammation (Abe et al, 2019; Erdal et al, 2017). Although their functions seem to overlap with each other, cytosolic DNA sensors exhibit great diversity in molecular structures, DNA-binding modes, and distribution in different kinds of cells, which are related to their biological effect preferences (Fig. 1, Table 1).

**Table 1:** Comparison of the main characteristics of major cytosolic DNA sensors.

Name	cGAS	IFI16	AIM2	ZBP1	DDX41
Cell type confirmed to express in CNS	Microglia (Ding et al, 2022; Li et al, 2021a). Astrocyte (Giordano et al, 2022; Jeffries and Marriott, 2017) Neuron (Welch et al, 2022).	Microglia (Cox et al, 2015; Jeffries et al, 2020). Astrocyte (Cox et al, 2015).	Microglia (Ma et al, 2021). Astrocyte (Barclay et al, 2022). Neuron (Lammert et al, 2020; Wu et al, 2016). T cells (Chou et al, 2021).	Microglia (Saada et al, 2022). Astrocyte (Jeffries et al, 2022). Neuron (Daniels et al, 2019)	Unclear, likely Microglia (Wang et al 2024; Tan et al, 2022).
Type of DNA that can bind	dsDNA (Civril et al, 2013) DNA:RNA hybrid (Mankan et al, 2014); >25 bp (Luecke et al, 2017); mostly B-DNA (Civril et al, 2013).	dsDNA/ssDNA; >70 bp (Jin et al, 2012b; Unterholzner et al, 2010); mostly B-DNA (Ni et al, 2016).	dsDNA; >70 bp (Jin et al, 2012b; Unterholzner et al, 2010); mostly B-DNA (Ni et al, 2016).	dsDNA (Jiao et al, 2020); >40 bp (Shen et al, 2014); Z-DNA and B-DNA (Ha et al, 2008; Jiao et al, 2020).	DsDNA/ssDNA (Singh et al, 2022), DNA:RNA hybrid (Mosler et al, 2021), nucleotide or cyclic dinucleotides (Jiang et al, 2017; Omura et al, 2016).
Subcellular Localization	Mostly cytoplasm (Sun et al, 2013); also nuclei (Jiang et al, 2019; Liu et al, 2018a).	Mostly nuclei (Dell’Oste et al, 2014); cytoplasm when simulated (Dell’Oste et al, 2014; Wang et al, 2019) Extracellular (Iannucci et al, 2020).	Mostly cytoplasm (Bosso and Kirchhoff, 2020; Hu et al, 2016).	Mostly cytoplasm (Kuriakose and Kanneganti, 2018; Zhang et al, 2020), especially stress granules (Deigendesch et al, 2006; Kuriakose and Kanneganti, 2018; Marcelo et al, 2021).	Mostly Nuclei (Parvatiyar et al, 2013); cytoplasm when stimulated (Singh et al, 2022).
Adaptor	STING (Sun et al, 2013; Yum et al, 2021). Chromatin fragments (Jiang et al, 2019; Liu et al, 2018a; Zhou et al, 2021b).	STING (Unterholzner et al, 2010) ASC (Chu et al, 2015). p53 (Fujiuchi et al, 2004; Kwak et al, 2003; Ouchi and Ouchi, 2008).	ASC (Chu et al, 2015; Morrone et al, 2015). DNA-PK (Ma et al, 2021; Wilson et al, 2015).	RIPK1 (Muendlein et al, 2021; Thapa et al, 2016). RIPK3 (Jiao et al, 2020; Lin et al, 2016). TBK1 (Kuriakose and Kanneganti, 2018). IRG1 (Daniels et al, 2019; Rebsamen et al, 2009).	STING (Zhang et al, 2011).
Activation mode	Dimerization (Zhang et al, 2014).	Oligomeriaztion (Cadena and Hur, 2019; Stratmann et al, 2015).	Oligomerization (Howard et al, 2022; Morrone et al, 2015).	Dimerization (Kuriakose and Kanneganti, 2018). Phosphorylation by TBK1 (Takaoka et al, 2007; Wang et al, 2008).	Phosphorylation by BTK (Lee et al, 2015).
Biological effect	IFN-I production (Sun et al, 2013; Yum et al, 2021). Autophagy (Gui et al, 2019). DNA repair blocking (Jiang et al, 2019; Liu et al, 2018a).	IFN-I production (Unterholzner et al, 2010) Autophagy (Liu et al, 2019). Checkpoint arrest (Fujiuchi et al, 2004; Kwak et al, 2003; Ouchi and Ouchi, 2008). Inflammasome (Stratmann et al, 2015). Viral DNA replication blocking (Bosso and Kirchhoff, 2020; Howard et al, 2022; Johnson et al, 2014; Roy et al, 2019).	Inflammasome (Barclay et al, 2022; Cunha et al, 2017; Gao et al, 2019; Hu et al, 2016; Stratmann et al, 2015). Pyroptosis (McKenzie et al, 2018).	IFN-I production (Takaoka et al, 2007; Wang et al, 2008). Apoptosis (Muendlein et al, 2021; Thapa et al, 2016). Pyroptosis (Jiao et al, 2020; Lin et al, 2016). NF-κB pathway activation. Inhibition of ZIKA virus (Rebsamen et al, 2009) replication (Daniels et al, 2019).	IFN-I production (Zhang et al, 2011). Autophagy (Liu et al, 2019).

### cGAS

Cyclic-GMP-AMP synthase (cGAS) is regarded as one of the most critical cytosolic DNA sensors in vertebrates. Human cGAS contains a conserved Mab1 domain that belongs to the nucleotidyltransferase (NTase) superfamily (Civril et al, 2013) and a flexible, lysine/arginine-rich N-terminus for promoting dimerization and strengthening cGAS-DNA binding (Tao et al, 2017). Mab1 is formed of two functional lobes and a connected spine, similar to oligoadenylate synthase 1(OAS1), a classic cytosolic RNA sensor (Hornung et al, 2014). During cGAS activation, lobe 2 binds to the minor groove of dsDNA with the help of the spine and a “Zinc-thumb” structure unique in cGAS (Civril et al, 2013). Combined with dsDNA, the conformation of lobe 1 transforms to be capable of catalyzing cyclic 2’-3’-GMP-AMP(cGAMP) with GTP and ATP (Civril et al, 2013). If no cytosolic dsDNA is detected, cGAS exists as a monomer regardless of its concentration but rapidly forms dimers as soon as cytosolic dsDNA appears, which indicates that dimerization is essential for normal functions of cGAS (Zhang et al, 2014). Generally, the two cGAS molecules in a dimer will bind to two different dsDNA chains, but they may share the same dsDNA chain if it is long enough, leading to ladder-like cGAS-DNA clusters and DNA condensation (Xie et al, 2019). As a conserved protein in vertebrates, human, and mice cGAS are homologous in most of the sequence (Margolis et al, 2017; Zhou et al, 2018). It has been reported, however, that two human-specific mutations in cGAS have strengthened its preference for long dsDNA chains, which may explain the much lower level of cGAMP in human cells compared to other vertebrates (Zhou et al, 2018).

dsDNA or DNA:RNA (Mankan et al, 2014) hybrids can fully combine with and activate cGAS, while ssDNA has much less affinity for cGAS (Deb et al, 2020; Kranzusch et al, 2013). dsRNA can bind robustly with cGAS in vitro but fails to activate it (Civril et al, 2013; Sun et al, 2013). As the primary activator in vivo, dsDNA requires no specific sequences but a certain length to match cGAS (Civril et al, 2013; Luecke et al, 2017). The lower the concentration of cytosolic dsDNA, the longer the dsDNA chain that is needed for cGAS activation. Using pcDNA3.1 as a template, the dsDNA chain around 25–40 bp needs at least 1 μg/ml to fulfill cGAS, while the chain around 800–2000 bp only requires 0.017 μg/ml, allowing cGAS to detect long dsDNA chains from pathogens(usually in Mbps) in small copy numbers at early the stage of infection (Luecke et al, 2017; Stetson and Medzhitov, 2006). Although cGAS prefers to combine with B-form DNA (Civril et al, 2013), other forms of DNA have been reported to be conjugated with cGAS (Herzner et al, 2015).

It has been well documented that cGAS mainly induces stimulators of interferon genes (STING) to trigger an innate immune response, including autophagy, type 1 interferon (IFN-I) production, and NF-κB pathway activation (Sun et al, 2013; Yum et al, 2021). However, STING-independent functions of cGAS have been discovered. Current research shows that cGAS can bind directly with chromatin DNA or poly ADP-ribose polymerase 1(PARP1) to prevent DNA damage repair by disrupting homologous recombination (Jiang et al, 2019; Liu et al, 2018a). cGAS can also support the function of IFI16 by strengthening its ability to bind with dsDNA and preventing it from degradation without stimulating STING (Orzalli et al, 2015).

In the CNS, cGAS is predominantly expressed in microglia, the resident myeloid cell in the brain (Deng et al, 2014; Ding et al, 2022; Li et al, 2021a). Dendritic cells (DCs) are also well-known to express cGAS, but whether these cells exist in the brain under physiological conditions remains controversial (Bulloch et al, 2008; McCauley et al, 2020). Peripheral monocytes can also activate the cGAS-STING signaling cascade when they infiltrate into the brain under pathological circumstances like herpes simplex virus 1(HSV-1) infection (Reinert et al, 2021). In addition to immune cells, cGAS is reported to be expressed in astrocytes in vitro (Jeffries and Marriott, 2017) and may have a central role in the rare genetic disease Aicardi-Goutières syndrome(AGS), which is characterized by abnormal IFN-I overproduction (Giordano et al, 2022). Also, cGAS is responsible for neuroinflammation in neurons with DNA double-strand break (DSB) burden during senescence (Welch et al, 2022). Notably, vascular cells, including endothelial cells and pericytes, have been reported in various studies to express cGAS in different tissues (Guo et al, 2020; Huang et al, 2020; Philipp et al, 2020). Furthermore, single-cell RNA sequencing of mouse brain vascular-associated cells has detected the expression of *Mb21d1* that encodes cGAS (He et al, 2018). However, conclusive evidence of cGAS expression in cranial endothelial cells or pericytes in vivo is still missing. As for subcellular location, cGAS is thought to be primarily located in the cytoplasm (Sun et al, 2013), but researchers have observed the localization of cGAS in nucleus. When DNA damage occurs, cGAS enters the nucleus and binds with chromatin fragments to suppress DNA repair and accelerate cellular senescence (Jiang et al, 2019; Zhou et al, 2021b).

### IFI16 and AIM2

Interferon-gamma induced protein 16 (IFI16, also known as P204 in mice) and absent in melanoma 2 protein (AIM2) both belong to the PYHIN family. They are comprised of an N-terminal pyrin domain (PYD) and a C-terminal Hematopoietic interferon-inducible nuclear protein with a 200-amino-acid repeat domain (HIN200) (Bosso and Kirchhoff, 2020). PYD, a member of the death domain fold (DDF) superfamily, is a component of various cell death-related proteins, including apoptosis-associated speck-like protein containing a C-terminal caspase recruitment domain (ASC), nucleotide oligomerization domain-like receptors (NLRPs) and Pyrin and hematopoietic interferon-inducible nuclear (PYHIN) (Chu et al, 2015). Through PYD-PYD interaction, both AIM2 and IFI16 can bind with ASC to form inflammasomes (Chu et al, 2015). HIN200 domains have three confirmed subtypes termed HIN A, B, and C, which are unique to the PYHIN family. IFI16 contains a HIN A and a HIN B domain, while AIM2 only has a single HIN C domain (Jin et al, 2012b). According to phylogenetic analyses, the HIN200 family originated from a common ancestral gene in mammals and differentiated into HIN C, B, and A in turn, all of which retained their abilities to bind DNA during evolution (Cridland et al, 2012). These three HIN domains contain two oligonucleotide/oligosaccharide binding (OB) folds to serve as DNA binding sites (Albrecht et al, 2005). While contacting dsDNA, both OB folds of the HIN C domain and the linker between them can form hydrogen bonds and van der Waals (vDW) contacts with the DNA phosphate backbone across the major and minor grooves. Although HIN B isolated from IFI16 has a similar ability to bind both strands of the dsDNA phosphate backbone like HIN C, binding residues of the HIN B domain have less exposed surface area in a complete IFI16 molecule, which may explain the weaker affinity of HIN B in IFI16 compared to HIN C in AIM2 (Jin et al, 2012b). On the other hand, HIN A only contacts the phosphate backbone of one DNA strand, therefore it has less affinity to DNA than HIN B and C, but gains the capacity to bind to ssDNA (Fan et al, 2021; Hurst et al, 2019; Ni et al, 2016; Unterholzner et al, 2010).

Interestingly, self-binding can occur between PYD and PYHIN domains to form self-restricted structures preventing spontaneous innate immune response in both IFI16 and AIM2 under normal circumstances (Jin et al, 2012b). Once free-DNA is detected, IFI16 and AIM2 initiate oligomerization to assemble filament-like structures along the DNA strands, which is essential for AIM2-mediated inflammasome formation and IFI16-mediated suppression of viral DNA transcription (Howard et al, 2022; Morrone et al, 2015). Of note, multiple IFI16 molecules can initiate oligomerization by tracking along the nucleosome-free DNA and interacting with each other (Stratmann et al, 2015). Howerver, whether they influence IFI16-dependent activation of STING remains unknown (Cadena and Hur, 2019).

It is generally considered that detection and combination of DNA with AIM2 or IFI16 does not rely on DNA sequence, according to its binding mechanism (Howard et al, 2022; Jin et al, 2012b; Ni et al, 2016). Similar to cGAS, AIM2 and IFI16 bind with DNA and trigger a downstream reaction in a length-dependent manner. Although the HIN200 domain shows an affinity with DNA as minimal as 10–20 bp, it cannot initiate inflammasome assembly or IFN-I production unless the DNA length reaches 70 bp, and it has an optimal effect when the DNA is longer than 200 bp (Antiochos et al, 2022; Jin et al, 2012b; Matyszewski et al, 2018; Unterholzner et al, 2010). The length-dependent sensing of DNA may serve as a potential mechanism for AIM2 and IFI16 to distinguish pathogenic DNA from host DNA since the dsDNA linker between two nucleosomes is about 20 to 30 bp in mammals (Stratmann et al, 2015). The affinity of the HIN200 domain binding with different configurations of DNA is comparable between Z-form and B-form DNA, but AIM2 and IFI16 predominantly bind with B-form DNA (Ni et al, 2016).

As mentioned above, IFI16 and AIM2 can both bind with DNA to unfold their autoinhibited structure composed of PYHIN and PYD, hence interacting with ASC with their PYD domain (Chu et al, 2015; Kerur et al, 2011; Matyszewski et al, 2018). Long pathogenic DNA provides a platform for multiple AIM2/IFI16-ASC complexes to oligomerize and assemble into inflammasomes (Stratmann et al, 2015). Cunha et al has also reported that the AIM2-ASC complex could trigger non-canonical NLRP3 inflammasome formation by recruiting non-cleaved caspase-1 to cause pore formation and K+ efflux (Cunha et al, 2017). Besides inflammasome formation, AIM2 has been found to directly bind with DNA-PK to inhibit the activation of protein kinase B (PKB, also known as AKT), resulting in colon tumor growth suppression and microglia-mediated neuroinflammation restriction in two individual studies (Ma et al, 2021; Wilson et al, 2015). Compared to AIM2, IFI16 has binding capacities for broader species of downstream molecules, the most crucial of which is STING. Instead of using cGAMP as a second messenger, DNA-carrying IFI16 can directly bind with STING to trigger the TBK1-IRF3 pathway (Unterholzner et al, 2010), although the structure and mechanism of the IFI16-STING interaction needs further investigation, as it can only be presumed that the unique HIN A domain is involved (Ni et al, 2016). Besides stimulating STING directly, IFI16 can regulate the cGAS-STING-TBK1-IRF3 axis by assisting dimerization and translocation of STING, TBK1 recruitment, production and function of cGAMP, and even cooperation with cGAS to sense cytosolic DNA (Almine et al, 2017; Jønsson et al, 2017). Conversely, a study focusing on porcine has revealed an unexpected role of IFI16 in inhibiting the cGAS-mediated immune effect by competitively binding with DNA and STING (Zheng et al, 2020). Moreover, a previous study illustrated the degradation of IFI16 by overexpressed STING, thus it can be speculated that a negative feedback network of among cytosolic DNA sensing pathway has emerged but is yet to be clarified (Li et al, 2019). IFI16 also acts as a dual regulator in p53-mediated cell checkpoint control: once IFI16 is knocked down, p53 can trigger cell cycle arrest by inducing p21 expression; however, under conditions of DNA-damage, p53 relies on IFI16 to initiate apoptosis (Fujiuchi et al, 2004; Kwak et al, 2003; Ouchi and Ouchi, 2008). IFI16 can exert antiviral effects not only by triggering innate immune response but also by forming oligomers in the viral DNA to interfere with transcription and recruit transcription inhibitors directly (Bosso and Kirchhoff, 2020; Howard et al, 2022; Johnson et al, 2014; Roy et al, 2019).

Various studies all confirm that AIM2 has a non-negligible expression in most types of cells in the CNS, including microglia, astrocyte, neuron, oligodendrocyte, brain endothelial cells, and infiltrating immune cells such as macrophages and T cells (Barclay et al, 2022; Chou et al, 2021; Lammert et al, 2020; Wang et al, 2019; Wu et al, 2016). IFI16 is expressed in fewer brain cell types, including microglia, astrocyte, and infiltrating macrophages (Cox et al, 2015; Jeffries et al, 2020). Due to the lack of nuclear localization signals (NLS), AIM2 is generally considered to not enter nucleus, although some research challenges this assumption as AIM2 aggregates have been found in nucleus for preventing DNA repair via interference of chromatin decompaction, and forming nuclear inflammasomes to induce inflammation and cell death in radiation-induced injury (Bosso and Kirchhoff, 2020; Hu et al, 2016; Jiang et al, 2021). On the contrary, NLS-containing IFI16 predominantly localizes in the nucleus and can be transported to the cytoplasm in cases of infection and injury (Dell’Oste et al, 2014; Wang et al, 2019). IFI16 has even been found to enhance lipopolysaccharide (LPS)-TLR4-mediated inflammation extracellularly (Iannucci et al, 2020).

### ZBP1

DNA-binding protein 1 (ZBP1), also known as DNA-dependent activator of IFN-regulatory factors (DAI), is a distinct cytosolic DNA sensor that mainly binds with Z-form DNA. ZBP1 consists of two Z-DNA binding domains (ZBD), two receptor-interacting protein homotypic interaction motifs (RHIM), and a C-terminal signal transduction area (Kuriakose and Kanneganti, 2018). The ZBD domains of ZBP1 belong to the Zα subtype of the ZBD family. Although significant differences in the DNA-binding residues have been observed in two ZBD domains of ZBP1, they share a similar connecting mode with DNA and are both indispensable for detecting cytosolic DNA, oligomerization, and recruiting signaling adaptors (Ha et al, 2008; Ha et al, 2006; Schwartz et al, 2001). Both receptor-interacting protein kinase 1(RIPK1) and RIPK3 can bind with ZBP1 via homotypic interactions of RHIM (Kuriakose and Kanneganti, 2018). Lastly, the C-terminus also plays a role in ZBP1-mediated IFN-I production by recruiting the TBK1-IRF3 complex (Takaoka et al, 2007). Like cGAS, ZBP1 also requires DNA-dependent dimerization to evoke its biological activity (Kuriakose and Kanneganti, 2018).

As named, ZBP1 explicitly targets Z-form double-stranded nucleic acids, i.e. both DNA and RNA (Jiao et al, 2020). But DNA sensing by ZBP1 is not conformation-dependent because its ZBDs both conserve the ability to transform B-DNA into Z-DNA (Ha et al, 2008). Like the cytosolic DNA sensors mentioned above, interaction of ZBP1 with DNA or RNA is also length-dependent, starting at 40 bp and reaching optimal effects at lengths longer than 100 bp (Shen et al, 2014; Wang et al, 2008).

RHIM and the C-terminus are the two individual binding sites in ZBP1 and initiate distinct signaling pathways under different conditions. The RHIM motif of ZBP1 has affinity for both RIPK1 or RIPK3; hence the activation site of ZBP1 depends on the competitive combination. In detail, two studies conducted by the Manolis lab demonstrate that ZBP1 activation leads to the recruitment of phosphorylated RIPK3 hence triggering MLKL-dependent necroptosis, which can be blocked via cleavages to the kinase domain of RIPK3 by RIPK1-recruiting caspase8 (Jiao et al, 2020; Lin et al, 2016). On the contrary, in influenza A virus infection, the effect of ZBP1 is dominated by RIPK1 instead of RIPK3, through inducing caspase8-mediated apoptosis in the absence of RIPK3 (Thapa et al, 2016). Muendlein et al, discovered similar cell apoptosis induced by a ZBP1-RIPK1-caspase8 axis upon stimulation with LPS instead of DNA or RNA. Upon LPS-induced ZBP1 activation, ZBP1-RIPK1 binds with TIR-domain-containing adapter-inducing interferon-β (TRIF), an adaptor protein that also binds with TLR4. As a result, LPS stimulation can transmit its signal to ZBP1 via TRIF (Muendlein et al, 2022; Muendlein et al, 2021). Interestingly, RIPK1 and RIPK3 also collaborate to trigger inflammatory response and cell death. The respective binding of RIPK1 and RIPK3 to the two RHIM motifs of ZBP1 can lead to NF-κB activation or induce immune responsive gene 1 (IRG1) expression to block ZIKA virus replication (Daniels et al, 2019; Rebsamen et al, 2009). The function of the C-terminus is more simple than the RHIM motif because it can only form a complex with TBK1 and IRF3. Upon nucleic acids stimulation, ZBP1 initiates phosphorylation by recruiting TBK1. The phosphorylated ZBP1 can augment recruitment of TBK1 and IRF3 to form an active complex, which allows TBK1 to phosphorylate sufficient IRF3 to trigger IFN-I production (Takaoka et al, 2007; Wang et al, 2008).

Expression of ZBP1 has been observed in neurons, microglia, and astrocytes (Daniels et al, 2019; Jeffries et al, 2022; Saada et al, 2022). Despite a few studies that have found evidence of its localization in the nucleus, especially under infection of influenza A virus, ZBP1 is commonly thought to be localized in the cytoplasm (Kuriakose and Kanneganti, 2018; Zhang et al, 2020). It is worth noting that cytosolic ZBP1 highly aggregates in stress granules, the dynamic cytosolic compartments induced by various stimuli including heat, oxidative stress, and radiation damage. Stress granules consist of a large amount of RNA (about 10–15% mRNA of the entire cell) and proteins that stabilize and regulate mRNA processing and translation, but no lipid membrane on the surface (Deigendesch et al, 2006; Kuriakose and Kanneganti, 2018; Marcelo et al, 2021). As the function of stress granules is not fully understood, the role of ZBP1 in these granules also remains vague (Marcelo et al, 2021).

### DDX41

DEAD-Box Helicase 41 (DDX41) is an RNA helicase and a member of the DEAD family of DExD/H proteins (Andrisani et al, 2022). DExD/H proteins contain nine characteristic conserved motifs, each of which acts differently in various types of proteins. These nine motifs in DDX41 can be divided into the DEAD domain and helicase domain, where the former functions to bind DNA and interact with STING while the latter separates hybridized DNA strands (Zhang et al, 2011). Unlike typical cytosolic DNA sensors that only detect large molecules like DNA, DDX41 shows affinity to nucleotides, including AMP, ADP, ATP, cyclic di-AMP, and cyclic di-GMP in different binding sites, among which dsDNA and cyclic dinucleotides share the same site (Jiang et al, 2017; Omura et al, 2016). Although no strict limit on DNA length is required for DDX41 sensing, there is a positive correlation between the DNA length and the biological effect of DDX41 (Zhang et al, 2011). Interestingly, DDX41 exerts a dual influence on the DNA helix: ATP-dependent unwinding from dsDNA to ssDNA and ATP-independent annealing from ssDNA to dsDNA, which means that DDX41 not only has the capacity to trigger cytosolic DNA-sensing pathways but also regulates the activity of cytosolic DNA sensors, particularly those that are unable to detect ssDNA, such as cGAS (Singh et al, 2022).

DDX41 mainly triggers STING-TBK1-IRF3-IFNβ signaling by directly binding with STING (Zhang et al, 2011). This binding requires phosphorylation by Bruton’s tyrosine kinase (BTK) (Lee et al, 2015) and is enhanced by c-di-GMP since both DDX41 and STING can simultaneously interact with this bacteria-derived nucleotide (Parvatiyar et al, 2013). It is worth mentioning that another two DExD/H proteins, DHX9 and DHX36 (DExH-Box Helicase 9 and 36), can selectively sense bacteria-characteristic CpG-A and CpG-B oligonucleotides, respectively, and trigger IFN-β production, suggesting they are also specialized cytosolic DNA sensors (Kim et al, 2010). Microglia in the brain has garnered significant attention in the research of DDX41, and it is believed that microglial DDX41 plays a role in neurodegenerative diseases such as ALS and Parkinson’s disease (Wang et al, 2024; Tan et al, 2022). However, more research is required to investigate the expression and function of DDX41 in other CNS cell types. Although recognized as a cytosolic DNA sensor, DDX41 has been found to accumulate in the nucleus during normal circumstances (Parvatiyar et al, 2013) and translocates to the cytoplasm under simulation by cytosolic DNA (Singh et al, 2022).

## STING: the reaction centre of cytosolic DNA sensing pathways

The stimulator of interferon genes (STING), also known as transmembrane protein 173 (TMEM173), is the most crucial adaptor molecule for cytosolic DNA sensors. It can bind with cyclic dinucleotides like c-di-GMP, c-di-AMP and cGAMP from cGAS and cytosolic DNA sensors like IFI16 and DDX41 to trigger various biological effects including IFN-I production, non-canonical NF-κB pathway activation, autophagy, and metabolic reprogramming, hence playing vital roles in various physical and pathological processes (Fig. 1).

## Structure and activating mechanism of STING

STING, located in the endoplasmic reticulum (ER), contains four transmembrane helices in the N-terminus followed by a C-terminal domain (CTD). The CTD is composed of a cytoplasmic ligand-binding domain (LBD) and a C-terminal terminal tail(CTT) that serves as a signaling domain (Burdette and Vance, 2013). Through hydrophobic interaction, STING forms a symmetric butterfly-like dimer in vivo as the LBD domain “wings” stretch towards different directions to leave a groove between that is responsible for ligand detection and accommodation. STING remains inactive by self-binding between CTT and LBD without stimuli. Once the signaling molecule fits into the groove, both LBD domains connect to the ligand, causing the groove to switch into “closed” mode and unwinding the CTT-LBD self-binding site to allow recruitment of adaptor molecules (Burdette and Vance, 2013; Ergun et al, 2019; Ouyang et al, 2012). In a ligand-binding state, STING dimers assemble into polymers via a disulfide bridge, which is also required for successful STING activation (Ergun et al, 2019).

## Ligands of STING

C-di-GMP is the first ligand identified for STING (Burdette et al, 2011). But c-di-GMP injection in *Drosophila* failed to induce antimicrobial effects, and c-di-GMP has a weak binding affinity and slight influence on the conformation of STING, suggesting that it is not likely the major ligand in vivo for STING (Cai et al, 2020a; Ergun et al, 2019). Compared to bacterial cyclic dinucleotides like c-di-GMP and c-di-AMP, cGAMP produced by cGAS shows much higher affinity and induces stronger activation of STING, and is considered as the most important endogenous activator of STING (Ergun et al, 2019; Guo et al, 2019). Although the structure of STING-cyclic-dinucleotide complexes has been well studied, the molecular basis of STING directly binding with cytosolic DNA sensors like IFI16 and DDX41 still needs further investigation.

## Functions of STING

STING was first identified as the trigger of innate immune response under stimulation from cyclic dinucleotides, as it enables massive IFN-I production to prevent microbial infection. However, recent studies have revealed other important biological functions of STING irrelevant to IFN-I and even immune response. Below we discuss the main functions of STING.

### STING-TBK1/IKKε-IRF3/NF-κB/STAT6 pathway

Tumor necrosis factor receptor-associated factor (TRAF) family member associated NF-κB activator (TANK)-binding kinase 1 (TBK1) and its homolog inhibitor of nuclear factor kappa-B kinase subunit epsilon (IKKε) are the most crucial downstream effectors of STING. Activated STING and TBK1 assemble a complex, followed by recruitment phosphorylation of transcription regulatory factors including interferon regulator 3 (IRF3), IKKε and Signal transducer and activator of transcription 6 (STAT6) (Chen et al, 2011). The structure of the STING-TBK1 complex has been well studied. In detail, inactivated TBK1 dimers and STING dimers are observed to have constant interaction even without ligand binding, and the interaction still exists in mutant STING lacking ligand-binding sites. But under the stimulation of cGAMP, the CTT of STING is released from self-binding mode with LBD in order to form a more solid bond with TBK1, which is essential for further activation of STING and TBK1. Then STING starts transportation from the ER to Golgi bodies and disulfide bridge-dependent polymerization, which offers a platform for more TBK1 recruitment and allows them to contact each other for transautophosphorylation. Phosphorylated TBK1 also induces phosphorylation of adjacent STING. The polymerization and phosphorylation of STING are sufficient prerequisites for the recruitment of IRF3, which will land in the CTT for dimerization and TBK1-induced phosphorylation (Ogawa et al, 2018; Tanaka and Chen, 2012; Zhang et al, 2019). The activated IRF3 then translocates to the nucleus, eventually leading to CXCL10, CCL5, and type 1 IFN expression (Yanai et al, 2018). The type 1 interferons (IFN-I) family, emcompassing IFN-α, IFN-β, IFN-ω, IFN-ɛ, and IFN-к, constitute the principle cytokines responding to most of the cytosolic DNA sensing pathways. IFN-I bind to IFN-α receptor (IFNAR), a characteristic shared by all members of the family, leading to similar biological effects in neurodegenerative disease, which will be discussed in later sections.

Despite activating IRF3 to trigger IFN-I production, the STING-TBK1/Ikkε complex is also involved in regulation of the NF-κB pathway as both TBK1 and Ikkε were initially discovered as the activators of the non-canonical TRAF2-TANK-NF-κB-inducing kinase (NIK)-NF-κB pathway, and as such are considered members of the non-canonical IkB kinase family (Nakanishi and Akira, 2000; Pomerantz and Baltimore, 1999). Since then, a considerable amount of research has reported that STING-TBK1 mediation can lead to NF-κB activation, and specific inhibition against it attenuates NF-κB-related pathological behavior (Cai et al, 2020b; Möller et al, 2020). But the roles of TBK1 and Ikkε in the NF-κB pathway have been challenged because they may act contrarily to their established roles from the past few decades. In immortalized dermal fibroblasts derived from patients with a loss-of-function mutation in *Tbk1*, STING-mediated IFN-I production is greatly reduced, but NF-κB-mediated IL-6 release remains nearly unchanged (Taft et al, 2021). In vitro assays using *Tbk1/Ikbke* knockout macrophages or specific TBK1/Ikkε inhibitor treatment also reveal a mild effect on STING-mediated NF-κB activation, indicating that TBK1/Ikkε is unnecessary in this process (Balka et al, 2020). Moreover, TBK1 was found to have the capacity to phosphorylate NIK in B cells, followed by ubiquitination-mediated NIK degradation and suppression of non-canonical NF-κB activation (Jin et al, 2012a). Taken together, the role of TBK1/Ikkε in the NF-κB pathway is yet to be fully clarified.

### STING-autophagy pathway

The link between STING and autophagy dates back to 2009, a year after STING was discovered when Saitoh et al, found STING colocalized with TBK1 as well as microtubule-associated protein 1A/1B-light chain 3 (LC3) and autophagy-related 9A (ATG9A) in ER-Golgi intermediate compartments (ERGIC) under dsDNA stimulation (Saitoh et al, 2009). Later research by Watson et al, found that the autophagy in response to cytosolic DNA was inhibited in *Sting1*^*-/-*^ bone-marrow-derived macrophages (BMDM) in vitro when infected by *M. tuberculosis*, which proved that cytosolic dsDNA-induced autophagy predominantly relies on STING (Watson et al, 2012). Since then, the STING-mediated autophagy pathway has been well studied. When stimulated by cGAMP, STING molecules start polymerization on the surface of ERGIC with the assistance of secretion-associated Ras-related 1 (SAR1) and SEC24C protein. The STING-bearing ERGIC subsequently translocates from ER to Golgi apparatus, which is essential for both autophagy activation and stimulation of the STING-TBK1-IRF3 axis. The STING-containing ERGIC recruits LC3, in which STING directly binds with LC3 and may orchestrate the LC3 lipidation in a WD repeat domain phosphoinositide-interacting protein 2 (WIPI2)- and ATG5-dependent mechanism (Gui et al, 2019; Liu et al, 2019). Although STING-mediated autophagy and TBK1 activation share the essential step of SAR1- and SEC24C-dependent puncta formation, and blockade of these proteins also prevents the phosphorylation of TBK1 and its downstream effects, it should be pointed out that STING triggers autophagy independently of the STING-TBK1-IRF3 axis (Gui et al, 2019). Mice with STING mutation of S365A (unable to bind with IRF3), L373A (unable to bind with TBK1), or ΔCTT (complete loss of CTT) all show no effect on STING-mediated autophagy, while ablation of WIPI2 impedes autophagy but does not alter the phosphorylation of TBK1 and IRF3 (Gui et al, 2019; Yum et al, 2021). In fact, the function of STING in triggering autophagy appears much earlier than its role in the TBK1-IRF3-IFNβ pathway in evolution, because STING from sea anemone *Nematostella vectensis* only induces autophagy but not interferons in response to stimulation by cGAMP (Gui et al, 2019). Among invertebrate animals, *Drosophila melanogaster* has a STING protein (referred to as dSTING) that can induce both autophagy and the production of antimicrobial peptides (Cai and Imler, 2021). Researchers have discovered dSTING can restrict Zika Virus (ZIKV) infection in the brain by promoting autophagy and activating the Relish-dependent IMD pathway. This pathway is a conserved NF-κB pathway found in insects, which has antiviral and anti-bacterial effects by inducing the production of diptericin, dSTING, Nazo, etc. (Goto et al, 2018; Liu et al, 2018b). Unlike vertebrate animals, dSTING in Drosophila can be activated by two forms of endogenous cyclic dinucleotides, namely 2′3′-cGAMP and 3′2′-cGAMP, These cyclic dinucleotides are actually produced by cGAS-like receptors(cGLRs) that recognize cytosolic dsRNA instead of dsDNA (Holleufer et al, 2021; Slavik et al, 2021).

### STING and metabolism

Increasing evidence has revealed the influence of STING on metabolism. Hasan et al, show that in a three prime repair exonuclease 1 knockout (*Trex*^*-/-*^) mouse model (which results in cytosolic DNA accumulation and STING overactivation), TBK1 induces metabolic disorder by suppressing mammalian target of rapamycin complex 1 (mTORC1) activity (Hasan et al, 2017). But some studies argue that TBK1 plays a positive role in mTORC1, and these contradictory consequences may be due to different TBK1-induced phosphorylation sites in mTORC1 under additional stimulation (Antonia et al, 2019; Bodur et al, 2018). The absence of STING can induce phosphorylation of adenosine monophosphate-activated protein kinase (AMPK), thus prompting the translocation of glucose transporter 4 (GLUT4) to the cell membrane and increasing insulin-dependent glucose uptake (Rong et al, 2022). STING also directly interacts with fatty acid desaturase 2 (FADS2) to restrain and maintain the balance between inflammation and lipid metabolism, but this process seems to be irrelevant to its activation by cytosolic DNA because similar changes are not observed while modulating cGAS instead of STING (Vila et al, 2022). Amlexanox, a specific inhibitor of TBK1 and IKKε, is found to rescue metabolic dysfunction in obesity, including overweight reversal, improvement of insulin resistance, and enhancement of adipose mobilization via upregulating uncoupling protein 1 (UCP1), therefore it is currently under phase II clinical trial for type II diabetes and obesity (Reilly et al, 2013). Taken together, metabolism is controlled by STING-mediated response in a non-canonical and two-sided way, although further studies are required for more insight.

### Regulation of cytosolic DNA sensor-STING pathway

As the regulator of the innate immune response induced by cytosolic DNA sensors, STING receives various kinds of inputs to amplify its effects for sufficient immune response against microbial invasion, or to restrict over-reaction to avoid autoimmune damage in post-infection and healthy circumstances. cGAS, IFI16, and STING are members of interferon-stimulated genes (ISGs). As they are able to induce strong production of IFN-I, a positive feedback loop for IFN-I pathway is built and produces a sufficient level of IFN-I to maintain an effective innate immune response to inhibit viral replication (Dawson and Trapani, 1995; Ma et al, 2015). But there are also other ISGs that serve as inhibitors of the positive feedback loops of the IFN-I pathway. Interferon-induced transmembrane protein3 (IFITM3) was discovered to bind to STING to ameliorate phosphorylation and activation by p-TBK1 in the late phase of DMXAA trigger (Motani and Kosako, 2020). Another ISG, TREX1, is a cytoplasmic 3’-5’ exonuclease that aims to degrade cytosolic DNA rapidly to control persistent innate immune responses. Its deficiency in humans leads to the accumulation of mislocalized DNA, over-activation of STING-related pathways and excessive IFN-I production, which cause a kind of rare inherited genetic disease called Aicardi-Goutieres syndrome (AGS) (Crow et al, 2006). Despite regulation by ISGs, activated STING can recruit the E3 ligase tripartite motif-containing protein 21 (TRIM21) to induce ubiquitination-dependent IFI16 and DDX41 degradation (Li et al, 2019; Zhang et al, 2013). As for the regulation of STING, cGAMP from cGAS can dephosphorylate AMPK to unchain it from unc-51-like autophagy-activating kinase 1 (ULK1), followed by ULK1-induced phosphorylation of suppressive sites in STING (Konno et al, 2013). TBK1 can phosphorylate p62 in an IRF3-dependent manner, resulting in p62-mediated autophagy and degradation of STING (Prabakaran et al, 2018). p62 also serves as the guider to lead AIM2 binding with TRIM11 to form an autophagosome for degradation (Liu et al, 2016). These mechanisms maintain the delicate balance between immune response and homeostasis. However, under persistent stimuli from diseases like neurodegeneration, such balance can be broken and lead to a disastrous cycle coordinated by continuous cell damage, DNA leakage, and over-stimulated cytosolic DNA sensing pathways.

## Cytosolic DNA sensors in pathological progress of neurodegenerative diseases

Abnormal activation of cytosolic DNA sensors has been found to be involved in a broad spectrum of diseases, including systemic lupus erythematosus (SLE) (Antiochos et al, 2022), atherosclerosis (Pham et al, 2021), and non-alcoholic fatty liver disease (NAFLD) (Luo et al, 2018). Given the fact that AGS, the genetic disorder related to abnormal activation of the cytosolic DNA sensing pathway, is characterized by severe encephalopathy, including neurodevelopmental decline and frequent seizures (Crow et al, 2006), it is conceivable that there is a tight relationship between cytosolic DNA sensors and CNS disorders. Here, five kinds of common neurodegenerative diseases as well as their research status with cytosolic DNA sensors are discussed.

### Alzheimer’s disease (AD)

**Figure 2:**
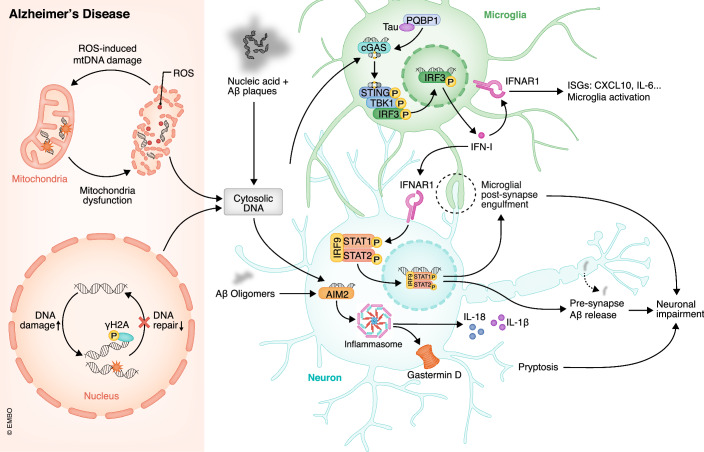
Alzheimer’s disease (AD) and cytosolic DNA sensors. There are several factors that contribute to the source of cytosolic DNA, including a decrease in nucleic DNA repair functions, mitochondrial reactive oxygen species (ROS) damage, and the presence of nucleic acid-containing Aβ plaques. In response to accumulation of cytosolic DNA and PQBP1-binding Tau protein, microglia initiate the activation of the cGAS-STING pathway and IFN-I production. Neurons suffer impairment from enhanced synapse engulfment by activated microglia, Aβ release by IFN-I, and inflammasome formation by direct cytosolic DNA sensing by AIM2.

AD is one of the world’s most widespread and troublesome neurodegenerative diseases, as it leads to irreversible cognitive impairment and certain death (Patterson, 2018). Amyloid-β protein plaques and abnormal phosphorylated tau protein that forms neurofibrillary tangles are characterized as pathological features and likely contribute to the development of AD (Breijyeh and Karaman, 2020). However, multiple research and clinical trials that target them have failed, urging scientists to find other possible suspects. Recently, abnormal activation of cytosolic DNA sensing pathways in progression of AD have raised interest, especially over-production of IFN-I (Fig. 2).

DNA damage is a hallmark in the early pathogenesis of AD. Staining of the γ-phosphorylated form of H2A histone family member X (γH2AX), a specific DSB marker, is greatly increased in neurons and astrocytes in postmortem brain tissues from patients diagnosed with AD and even mild cognitive impairment (MCI) (Shanbhag et al, 2019). Increased γH2AX staining is further confirmed in a classical AD murine model 5xFAD, as it can be found as early as in 5-month-old mice before typical Aβ plaques and neurofibrillary tangles are formed (Thadathil et al, 2021). Damaged nuclear DNA can drop from chromosomes and become free DNA fragments. During mitosis, the nuclear membrane disintegrates and DNA fragments cannot be controlled by the spindle apparatus, hence spontaneously assembling with the fragments of the former nuclear membrane to form a micronucleus(MN) (Fenech et al, 2011). MN is unstable and fragile and its breakdown allows nuclear DNA to be released into the cytoplasm and quickly detected by cytosolic DNA sensors such as cGAS (Mackenzie et al, 2017). MN is identified as a potential early-stage biomarker for AD, because it is not only found in the CNS but also in peripheral cells such as lymphocytes, fibroblasts, and even oral mucosal epithelial cells in AD patients (Migliore et al, 2011), indicating that cytosolic DNA leakage is an early and frequent event for AD. Mitochondrial DNA (mtDNA) damage also occurs in AD patients and murine models with much higher frequency and more catastrophic influence. Mitochondria are the center of aerobic respiration and lack complete DNA repair mechanisms, therefore they suffer higher risks of mtDNA damage and reactive oxygen species (ROS) production, resulting in a vicious oxidative stress-DNA damage cycle (Lin et al, 2020; Mao and Reddy, 2011). Along with increased DNA damage, DNA repair mechanisms are significantly weakened in AD. Expression of 8-oxoguanine DNA glycosylase-1 (OGG1), Breast cancer gene 1(BRCA1), PARP1, and DNA-PKcs, as representatives for basic excision repair (BER), homologous repair (HR), and non-homologous end joining (NHEJ), respectively, are reported to decrease in AD due to aggravating oxidative stress (Lin et al, 2020; Pao et al, 2020). Another potential source of cytosolic DNA is extracellular DNA released by dead cells since it can scaffold Aβ protein to form nucleic acids-containing plaques, which can be uptaken by microglia (Braun et al, 2011; Liu and Zhang, 2011).

Among cytosolic DNA sensors, cGAS and AIM2 have been found to have a solid link to AD. Expression of cGAS in 5xFAD mice significantly increases at the age of 7 months, reasonably later than emerging DNA damage accumulation at 5 months as introduced above (Hou et al, 2021). The latest research by Xie et al, reveals that knockout of cGAS or pharmaceutical inhibition of STING by H-151 both rescue pathological features in 5xFAD mice, including cognitive impairment, amyloid-β pathology, and neuroinflammation (Xie et al, 2023). But it should be noted that H-151 has been proven to exert off-target effects irrelevant to STING, so the benefit from H-151 may not only be dependent on STING inhibition (Hong et al, 2021). Interestingly, cGAS also experiences DNA-independent activation in tau-related pathology. It can directly bind with polyglutamine-binding protein 1(PQBP1) that bridges tau protein and cGAS via its WW domain (Jin et al, 2021; Yoh et al, 2015). However, another study conducted by Udeochu et al, reveals the activation of cGAS through tau induction in an mtDNA-dependent manner, inconsistent with the proposed tau-PQBP1-cGAS complex. In addition, the involvement of cGAS-STING in tau-related cognitive decline has been identified, resulting in the inhibition of neuronal expression of myocyte-specific enhancer factor 2C(MEF2C) expression (Udeochu et al, 2023). cGAS primarily exerts its influence on AD via the cGAS-STING-TBK1-IRF3-IFN-I pathway. IFN-I and ISGs have been found to be elevated in a 6.5-month-old AD mouse model, mainly affecting microglia and neurons (Hou et al, 2021; Roy et al, 2020). Using an 11-month-old MxG:5xFAD hybrid mouse, Roy et al, found 98.5% of plaques were colocalized with nucleic acids, and almost 80% of microglia near Aβ plaques expressed a typical ISG molecule Mx1 (MX dynamin-like GTPase 1). They also highlighted the morphology and gene expression changes of microglia in response to IFN-I, including extended process, inflated soma, enhancement of phagocytosis, and upregulation of pro-inflammatory genes (*Cd68*, *Clec7a*, *Il1b*, *Il-6*, and *Tnf*), indicating that IFN-I-induced alteration of microglia is universal and profound in AD (Moore et al, 2020; Roy et al, 2022; Roy et al, 2020). Notably, the extended microglial process engulfed the post-synaptic protein PSD95, leading to its degradation and progressive synapse loss in AD. Both *Ifnar1* deletion and IFNAR blockade by IFNAR1 neutralizing antibody rescue microglial phenotypes, synapse loss, inflammation, neuritic tau accumulation, and cognitive dysfunction. But microglial-specific *Ifnar1* deletion only ameliorates post-synaptic loss, while neural-specific *Ifnar1* deletion not only improves pre-synaptic loss but also decreases Aβ plaque burden, meaning that IFN-I inhibition attenuates AD pathologies by preventing microglia-mediated inflammation and its phagocytosis to postsynaptic structure, as well as neuron-mediated Aβ release from pre-synaptic membrane (Roy et al, 2022). In addition, microglial phagocytosis is not universally dampened under IFN-I inhibition, as *Ifnar1* knockout microglia reveal stronger but preferential phagocytosis towards Aβ_1-42_ (Moore et al, 2020).

AIM2, on the other hand, alters AD via inflammasomes instead of IFN-I. AIM2 is upregulated in APP/PS1 mice as early as 4 months of age and possesses affinities not just for cytosolic DNA but also for oligomeric Aβ (Cao et al, 2021; Chen et al, 2019). AIM2-ASC inflammasome formation promotes cleavage of pro-IL1-β, pro-IL-18, and Gasdermin D (GSDMD) via recruitment of caspase 1, thereby contributing to deteriorating neuroinflammation and pyroptosis in AD (Rui et al, 2021). Unexpectedly, *Aim2* deletion in 5xFAD mice attenuates Aβ load and microglia activation but does not improve the cognitive impairment and secretion of IL-1β and IL-18, while *Aim2* deletion in APP/PS1 mice rescues all of them. Such differences may be ascribed to the insufficiency of *Aim2* deletion to erase earlier and stronger Aβ plaques load in 5xFAD mice (Cao et al, 2021; Chen et al, 2019; Wu et al, 2017). These paradoxical results suggest the role of AIM2 in AD is still far from comprehensive elucidation.

It has to be noted that RIPK1-induced necroptosis is regarded as an important feature in the pathogenesis of AD. However, there is still a lack of research to clarify whether ZBP1 is involved in necroptosis in AD because multiple molecules have the ability to activate RIPK1 (Li et al, 2021b; Mathys et al, 2017).

### Parkinson’s disease (PD)

**Figure 3:**
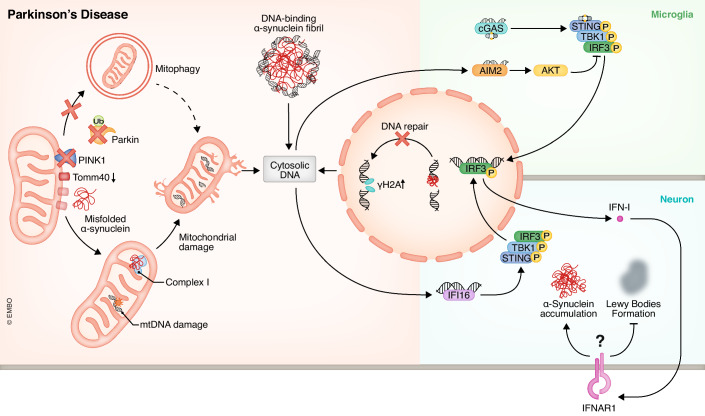
Parkinson’s disease (PD) and cytosolic DNA sensors. In PD, mitophagy dysfunction due to abnormal PINK1 or parkin expression results in mtDNA damage and leakage to the cytoplasm. Misfolded α-synuclein leads to cytosolic DNA accumulation by damaging mitochondria and directly binding with DNA. The response to cytosolic DNA in PD primarily involves activation of the cGAS-STING pathway and IFN-I production. However, there is ongoing debate regarding whether IFN-I is beneficial or detrimental to PD.

PD is a neurodegenerative disease characterized by tremors, rigidity, hypokinesia, and gait abnormality. It mainly affects dopaminergic neurons in the substantia nigra (SN), causing progressive abnormal α-synuclein aggregation, Lewy body formation, and irreversible neuron loss (Jankovic and Tan, 2020). The pathogenesis of PD remains unknown, and no effective treatment are available. Misfolded α-synuclein is widely regarded as the key factor in PD, and recent research has found that abnormal cytosolic DNA sensing pathways prompt its accumulation and pathological effects (Fig. 3).

mtDNA is the major origin of cytosolic DNA in PD. In 1999, an increase in cytosolic 8-hydroxyguanosine (an oxidation-induced damaged product of nuclear acids) was observed in SN tissue from PD patients, and it was confirmed that it originated from RNA and mitochondria (Zhang et al, 1999). Significant mtDNA deletion and respiratory chain dysfunction have been more recently discovered in SN neurons, indicating apparent mitochondrial damage and mtDNA leakage in PD (Bender et al, 2006). Misfolded α-synuclein can directly bind with mitochondria under the deficit of Translocase of outer mitochondrial membrane 40 (TOM40) and cause mitochondria to collapse along with ROS production and mtDNA release (Bender et al, 2013). As the most important cause of early-onset PD, loss-of-function mutations of PINK1 and/or parkin also lead to mtDNA leakage due to interruption of the mitophagy-lysosome pathway and mtDNA degradation (Matsui et al, 2021; Sliter et al, 2018). Besides mtDNA leakage, Schaser et al, discovered α-synuclein is a DNA-binding protein. Its accumulation can interfere with nuclear DNA repair, resulting in nuclear DNA damage and release (Schaser et al, 2019).

Both cGAS and IFI16 contribute to STING-mediated neuroinflammation in PD. Primary microglia incubated with α-synuclein-preformed fibril (α-Syn PFF) show apparent DSB labeling by γH2A, strong activation of the cGAS-STING pathway, and intense pro-inflammatory response in vitro, and bilateral striatal injection of α-Syn PFF to wildtype mice successfully reproduces the results in vivo. Deletion of STING attenuates microglial phenotype, α-synuclein fibril burden, and motor deficits in mice (Hinkle et al, 2022). Macrophages with *Lrrk2* mutation, which is common in patients with inherited and sporadic PD, express more cGAS than normal macrophages and exhibit similar phenotypes to microglia incubated with α-Syn PFF (Wallings et al, 2020). STING deletion also rescues PD-like pathological features and behavior in *Pink1*^*-/-*^ and *Prkn*^*-/-*^ mice with exhaustive exercise. Accmulation of cytosolic DNA, IFN-I release, and neuronal loss are also observed in the zebrafish model of PD, but the dominating cytosolic DNA sensor becomes IFI16 instead of cGAS in humans and mice (Matsui et al, 2021; Sliter et al, 2018). Taken together, STING-mediated neuroinflammation activated by cytosolic DNA sensors plays a general role in PD regardless of different pathological conditions.

The IFN-I impact on PD is indistinct and controversial. Main et al, found that the blockade of IFNAR1 rescues neuroinflammation and dopaminergic neuron loss in 1-methyl-4-phenyl-1,2,3,6-tetrahydropyridine (MPTP)-induced PD-like mice (Main et al, 2016), and offer a possible link between IFN-I toxicity and mitochondrial dysfunction since they find IFNAR1 blockade protects neuron from rotenone, a classical complex 1 inhibitor in another study (Main et al, 2017). On the contrary, other research argues that IFN-β deficiency leads to PD-like behavior due to apparent motor abnormalities in balance, grasp, and coordination in *Ifnb*^*-/-*^ mice. *Ifnb*^*-/-*^ mice also exhibit dopaminergic neuron loss in SN, α-synuclein accumulation, and Lewy body formation. Because the PD-like pathologies in *Ifnb*^*-/-*^ mice start at 3 months old and progress continuously, IFN-β1 is important to maintain neuronal development and survival throughout the lifetime (Ejlerskov et al, 2015).

AIM2 and ZBP1 are also reported to be involved in the pathogenesis of PD. Instead of activating the inflammasome pathway, AIM2 negatively regulates microglial inflammation via its inhibition of the cGAS-STING pathway by preventing DNA-PK-AKT3-induced phosphorylation of IRF3 that is in synergy with TBK1 (Ma et al, 2021; Rui et al, 2022). Both TNFα and the transcriptional factor IRF1 are upregulated in brain tissue from PD patients. Upon TNFα stimulation of neurons, IRF1 translocates into the nucleus and binds to the ZBP1 promoter to activate ZBP1 transcription. Overexpressed ZBP1 triggers cell death mainly through caspase3-mediated apoptosis and partly caspase1-mediated necroptosis, but whether ZBP1 activation also relies on cytosolic DNA remains unknown (Kuriakose et al, 2018).

### Amyotrophic lateral sclerosis (ALS)

ALS is the most common motor neuron disease that causes progressive cell death of both upper and lower motor neurons, resulting in voluntary muscle spasticity, twitching, myasthenia, and atrophy. Most patients become paralyzed within a few years and die of respiratory failure (Kiernan et al, 2011). Even though the minority of inherited ALS is linked to specific mutations in e.g., SOD1 or C9orf72, the pathogenesis of the majority (about 90%) of sporadic ALS remains unknown, and no successful cure has been found (Hobson and McDermott, 2016).

**Figure 4:**
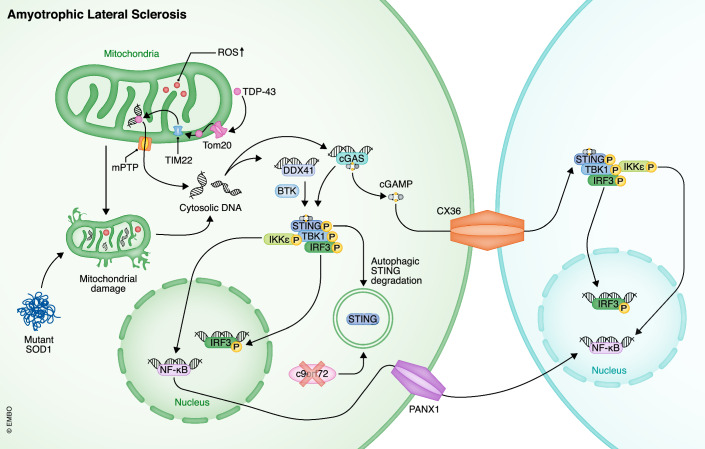
Amyotrophic Lateral Sclerosis (ALS) and cytosolic DNA sensors. Mitochondria are the main origin of cytosolic DNA in ALS due to increased oxidative stress caused by abnormal TDP-43 and mutant SOD1 leading to mitochondrial dysfunction. The release of cytosolic DNA further triggers the cGAS-STING pathway to exert an inflammatory response characterized by the production of IFN-I and activation of NF-κB activation. These effects can be propagated to neighboring cells through the transportation of cGAMP and NF-κB components via CX36 and PANX1, respectively.

Mitochondrial dysfunction has long been identified as a hallmark of ALS. In ALS, various abnormal molecules contribute to mitochondria damage, and all result in mtDNA leakage and cytosolic DNA sensing activation (Fig. 4). Superoxide dismutase 1 (SOD1), the most important factor in ALS, is located in mitochondria and functions to eliminate ROS. SOD1 loss-of-function mutations lead to increases in oxidative stress and abnormal SOD1 misplacement and aggregation, resulting in mitochondrial dysfunction and mtDNA damage (Tafuri et al, 2015). Using mitochondrial permeability transition pores (mPTP) as tunnels, mtDNA and mt(DNA:RNA) hybrids were shown to be able to escape from mitochondria and can be detected by cGAS and DDX41, respectively. Interestingly, the STING signaling pathway is still be activated in *cgas*^-/-^ cells if they are co-cultured with SOD^mut^ microglia, suggesting SOD1^mut^ microglia are able to transfer pro-inflammatory signals to bystander cells even without cGAS. Specifically, SOD1^mut^ microglia can activate non-immune cells like neurons and astrocytes by delivering cGAMP through gap junctions formed by connexin-36 and activated NF-κB components through PANX1 channels (Tan et al, 2022). Besides SOD1, the other two common pathogenic molecules, Mutant TAR DNA-binding protein 43 (TDP-43) and chromosome 9 open reading frame 72 (C9orf72) both induce neuroinflammation in a STING-dependent manner. Mutant TDP-43 in ALS accumulates in cells and is able to shuttle between mitochondria and cytoplasm: it can enter mitochondria via TOM20 in the outer membrane and TIM22 in the inner membrane, then open an mPTP-mediated tunnel to release mtDNA (Paolicelli et al, 2017; Yu et al, 2020). In CNS, C9orf72 mutation mainly affects microglia. Its loss-of-function mutation interrupts autophagy-mediated STING degradation, which allows persistent retention and activation of STING in late endosomes, hence inducing IFN-β production, DC development, and T-cell infiltration (McCauley et al, 2020). Neuroinflammation is triggered and propagated by the cGAS/DDX41-STING pathway in various ALS models via a different molecular mechanism, supporting its irreplaceable role in pathogenesis and possible treatment target in ALS.

### Aging

Rapid aging of the population causes a heavy social burden as it is a critical driver of various diseases, especially neurodegenerative diseases. Although the origins of senescence are complicated, upregulated levels of various cytosolic DNA sensors in brain samples from elderly people have reflected their importance in aging progress (Wang et al, 2016; Yu et al, 2022). DNA damage accumulation and continuous low-grade inflammation, as two of the most important hallmarks of aging, are both greatly relative to cytosolic DNA sensing pathways (López-Otín et al, 2023; Zhao et al, 2023). Cytosolic DNA sensors like cGAS and IFI16 can directly bind with chromatin DNA to prevent DNA repair (Ka et al, 2021; Liu et al, 2018a; Yang et al, 2017), hence certainly contributing to DNA damage accumulation in cell senescence. DNA damage accumulation furthermore serves as ample sources of micronuclei, which is sufficient to trigger persistent inflammation via cytosolic DNA sensing pathways (Bolognesi et al, 1999; Zhao et al, 2023). Despite micronuclei, nuclear envelope dysfunction is also partly responsible for nuclear DNA leakage to the cytoplasm. Hanna et al, demonstrated how decline of YAP/TAZ mechanosignalling during aging leads to cGAS-STING activation by disrupting the expression of lamin B1 and actin-related protein 2 (ACTR2), both of which are essential for nuclear envelope integrity (Sladitschek-Martens et al, 2022). Incomplete autophagy of damaged DNA exported from the nucleus also leads to abnormal activation of cytosolic DNA sensing pathways in senescent cells (Lan et al, 2019). Another origin of cytosolic DNA leakage is mitochondria. Gulen et al found that mitochondria-derived DNA leakage in microglia from the aged brain could stimulate the cGAS-STING pathway, triggering expression of the IFN-I gene, leading to microglia activation, neuroinflammation and accelerating cognitive impairment, which can be restored by either pharmaceutical STING blockage using H-151 or genetic *sting1* deletion in mouse (Gulen et al, 2023). Again, it should be kept in mind that the effectiveness of H-151 may not fully come from its inhibition of STING due to its off-target effects (Hong et al, 2021). However, it was shown to be effective in reversing neuronal toxicity from senescent BV2 cells by TNF-alpha antibody instead of IFNAR1 antibody, which challenges results from another study that genetic IFNAR1 deletion can attenuate senescence-derived microglial phenotype changes, neuronal loss, and neurodegenerative disease progression (Nazmi et al, 2019). Furthermore, whether cytosolic DNA sensors always perform detrimentally in ageing is also controversial. cGAS has been found capable of preventing cellular replicative senescence by binding with short telomeres to prevent chromosomal fusion (Li et al, 2022). ZBP1 not only assists telomere maintenance by localizing telomeric repeat-binding factor 2 (TRF2) correctly, but also recruits and activates ataxia-telangiectasia mutated serine/threonine kinase (ATM) for proper repair of DNA damage (Radak and Fallahi, 2023). Taken together, cytosolic DNA sensors play vital roles in aging and neurodegenerative disease, but their particular influences and mechanisms are yet to be elucidated.

### Multiple sclerosis (MS)

MS is the most prevalent autoimmune disorder affecting the CNS, with symptoms varying from progressive and relapsing paresthesia, dyskinesia, and parousia (Kister et al, 2013). No complete cure has been found for MS, and almost half of the patients eventually develop a steady progression of symptoms called secondary-progressive MS, even under sufficient medical care and immunotherapies (Cree et al, 2021). MS is characterized by demyelination, peripheral immune cell infiltration, and neuroinflammation spreading from the hemisphere to the cervical spinal cord (Lassmann et al, 2012). The pathogenesis of MS remains unknown, but scientists have found that experimental autoimmune encephalomyelitis (EAE), an animal model of neurological autoimmune disorder induced by injection of myelin components like MOG or MBP into rodents, was able to simulate the pathological features of MS and the board disease spectrum of autoimmune diseases in CNS (Constantinescu et al, 2011).

Although no study has tried to detect cytosolic DNA in brain or spinal cord tissues from MS patients, multiple pieces of evidence hint at its existence: oxidative stress is greatly enhanced in MS, causing mitochondrial dysfunction and mtDNA damage (Lu et al, 2000), which can be detected in the cerebral spinal fluid (CSF) (Varhaug et al, 2017); infiltrating B cells from the CSF and lesion sites mainly secrete anti-DNA antibodies in MS patients (Williamson et al, 2001); AIM2 is upregulated in MS (McKenzie et al, 2018); furthermore, DNA copies of Varicella zoster virus (VZV) dramatically increase during relapse periods of MS (Sotelo and Corona, 2011). As for EAE, the existence of cytosolic DNA has been confirmed (Mohamed et al, 2015; Wang et al, 2021). Deletion of C9orf72, a protein discussed above whose deficiency can induce mtDNA leakage, raises the susceptibility to EAE (McCauley et al, 2020).

The roles of DNA sensing pathways in MS and EAE remain controversial since IFN-β has long been recommended as the first-line treatment for MS and other neural autoimmune diseases (Rice and Ebers, 1998). The mechanism underlying its benefit is still not understood but may be related to suppressing T cell activation, upregulating anti-inflammatory cytokine (IL-4, IL-10, etc.), and inducing neural stem cell differentiation to oligodendrocytes for repairment (Hojati et al, 2016). As a reasonable speculation, triggering endogenous IFN-β production via cytosolic DNA sensor would be a potential strategy for treating MS and EAE, while blocking endogenous IFN-β production may exacerbate symptoms of MS and EAE. Indeed, in the murine EAE model, activation of the cGAS-STING pathway using cGAMP or DNA nanoparticles leads to improvement of progressive muscle weakness by promoting IL-10 and IL-27 secretion. However, this benefit is completely lost in STING knockout mice, indicating that exogenous DNA or cGAMP can improve MS and EAE in a STING-dependent manner (Johnson et al, 2021; Mohamed et al, 2015). Surprisingly, blocking the cGAS-STING pathway also improves MS and EAE, implying that the cGAS-STING pathway is detrimental in an IFN-β-irrelevant manner. Two individual studies have reported that the administration of Amlexanox alleviates EAE severity by directly suppressing TBK1-dependent activation and migration of memory and naive T cells, or by indirectly interrupting T cell recruitment and activation through inhibiting the maturation of DC cells (Quan et al, 2019; Yu et al, 2015). Research using the *cgas*^-/-^ mouse further demonstrates that ablation of the cGAS-STING pathway in DC cells can weaken the Th1/Th17 response to improve EAE (Mittal, 2021).

As mentioned above, the expression of AIM2 is increased in MS and EAE. Considering it is the only cytosolic DNA sensor that is not related to STING or IFN-β, it may have a distinct influence on MS and EAE. In fact, as expected, the upregulation of AIM2 is correlated with increasing caspase-1-induced inflammasome formation and pyroptosis (McKenzie et al, 2018). But Ma et al, have challenged the proposed harmful role of AIM2 in MS and EAE as they found the *Aim2*^*-/-*^ mouse presented strengthened microglial activation and peripheral immune cell infiltration, enhanced neuroinflammation, and worse pathological outcome, which is surprisingly due to alleviation of the cGAS-STING pathway (Ma et al, 2021). In conclusion, the impact of cytosolic DNA sensors in MS and EAE is still complicated and controversial. Further studies are needed to clarify their relations.

## Conclusion

Since ZBP1 was first identified in 2005, numerous cytosolic DNA sensors have been identified to be involved in various biological processes, including innate immune response, DNA repair, cell death, autophagy, and metabolism. Despite varying structures, DNA-binding modes, and cellular locations, cytosolic DNA sensors all have influences on innate immune response, particularly on STING activation and IFN-I production. Meanwhile, they still hold distinct functions and form complicated regulation networks that are far from clear elucidation. A large number of contradictory studies on their roles in the CNS also indicate that future research is needed to fully understand these molecules.

Various cytosolic DNA sensors often co-exist in the same cell, responding selectively according to different stimuli and playing different roles in physical or pathological processes. Such functional preference may be due to the crosstalk between different cytosolic DNA sensors. It has been already discussed above how DDX41 controls the activation of cGAS by transforming DNA between single-strand and double-strands (Singh et al, 2022), and inhibition of the STING-TBK1 pathway by microglial AIM2 (Rui et al, 2022), illustrating both positive and negative regulatory functions of cytosolic DNA sensors. But not all of the interactions between different kinds of cytosolic DNA sensors have been clarified, for example, the herpes simplex virus (HSV-1) infection activates cytosolic DNA sensors differentially according to cell type: cooperation of cGAS and IFI16 fully triggers an inflammatory response in human foreskin fibroblasts(HFF) (Orzalli et al, 2015), but neither of them is responsible for microglial antiviral response in HSV-1 infection (Jeffries et al, 2020); astrocytes with HSV-1, additionally, mainly undergo necroptosis and apoptosis mediated by ZBP1 (Jeffries et al, 2022). The mechanisms of these selective activations of different cytosolic DNA sensing pathways are still vague.

The evolution of cytosolic DNA sensors is another less studied but interesting question. Because they are members of the most primitive immune defense mechanisms in eukaryotes, cytosolic DNA sensors have accumulated apparent and complicated molecular discrepancies over time. Despite the sensitivity of cGAS (Zhou et al, 2018), differentiation of the PHYIN protein family (Cridland et al, 2012), and functional expansion of STING (Liu et al, 2019) mentioned above, the potential effects of evolutionary differences are still an open question. Compared to human IFI16, the murine homologue p204 comprises a serine/threonine (S/T)—a rich region between PYD and HIN200 domain (Liao et al, 2011). It is well-known that the S/T-rich region frequently serves as a modification site including phosphorylation and glycosylation, therefore murine p204 may possess unique functions and regulatory patterns that are not found in human homologue IFI16 (McCubrey et al, 2000).

Mechanistic studies of cytosolic DNA sensors and their pathways have grown rapidly in the past decade, but development of medicines and clinical trials are still at an early stage. Although many inhibitors have been successful in animal experiments (Domizio et al, 2022; Hinkle et al, 2022; Paolicelli et al, 2017; Vincent et al, 2017; Xie et al, 2023), so far virtually none have been tested in clinical trials until now, nor have they been tested for therapeutic effectiveness in neurodegenerative diseases. Amlexanox, an FDA-approved TBK1/IKKε inhibitor initially designed to treat asthma and dental ulcers, has now been found to be valuable for ameliorating inflammatory response in some diseases, including autoimmune encephalitis and sporadic aortic aneurysm and dissection (AAD), making it a candidate for clinical use in targeting the cytosolic DNA sensor-STING pathway (Luo et al, 2020; Quan et al, 2019). Anifrolumab, an IFNAR1 monoclonal antibody that recently received FDA approval to treat SLE, is another potential drug to be used against the cytosolic DNA sensing pathway in neurodegenerative diseases since symptoms of SLE include significant neurological dysfunction (Morand et al, 2020). However, effective therapies are still sought after, particularly regulators of specific cytosolic DNA sensors or STING.

Besides cytosolic DNA sensors, cytosolic dsRNA sensors are also important components of innate immune defense against viruses. Their aberrant activation is responsible for the development of various diseases. Cytosolic dsRNA sensors can be classified into several main classes: RIG-I-like receptors (RLRs), protein Kinase R (PKR), oligoadenylate synthases (OASes), adenosine deaminases acting on RNA (ADARs), RNA interference pathway (including Drosha, Dicer and Argonautes), PACT, and TRBP (Hur, 2019). These sensors are primarily involved in RNA editing processes such as splicing, degradation, and base substitution, ultimately rendering the viral RNA silenced (Eisenberg and Levanon, 2018; Hornung et al, 2014). However, PKR and RLRs, which encompass retinoic acid-inducible gene I (RIG-I) and melanoma differentiation-associated protein 5 (MDA5), do not participate in RNA editing. The presence of cytosolic dsRNA has been reported in various neurodegenerative diseases. In an ALS model with TDP-43 knockdown, mtRNA leakage led to RIG-I/MDA5 activation (Milstead et al, 2023). Mutations of tyrosine nonreceptor kinase-2 (TNK2) have been shown to induce PD both in human and animal models by enhancing dsRNA import (Nourse Jr et al, 2023). In AD, the tau protein has been implicated in the induction of dsRNA transcription from retrotransposons and the upregulation of genes involved in RNA-sensing pathways (Ochoa et al, 2023; Rexach et al, 2020). Juan et al, confirmed elevated expression of RLRs in AD and even MCI patients (de Rivero Vaccari et al, 2014). Similarly to cytosolic DNA sensors, dsRNA sensors bind to the mitochondrial antiviral signaling protein (MAVS) to induce inflammasome formation through recruiting NLRP3 and activation IFN-I via a TBK1/IKKε-IRF3/IRF7/NF-κB axis (Rehwinkel and Gack, 2020; Subramanian et al, 2013). Beyond that, multiple cytosolic DNA sensors share homologous sequences and similar structures with RNA sensors, such as the Mab-1 domain in cGAS and OAS1 (Hornung et al, 2014), the helicase domain in DDX41, RIG-I, and MDA5 (Kato et al, 2021), and the ZBD domain in ZBP1 and ADAR1 (Ha et al, 2008). cGAS, ZBP1, and DDX41 retain the ability to bind RNA or DNA:RNA hybrids (Jiao et al, 2020; Mankan et al, 2014; Mosler et al, 2021). Therefore, exploring whether cytosolic DNA and RNA sensors interact with each other in neurodegenerative diseases and understanding the biological mechanism behind them will be intriguing.

It is important to note that DNA sensors may contribute to other CNS pathologies, such as strokes, physical or chemical brain injury, and CNS infections. To wrap up, the roles of cytosolic DNA sensors in the CNS are currently not well understood. More studies are necessary to clarify their molecular characteristics, specific expressions in different cells, interaction networks, and crucial impact on the development of brain diseases. Only by doing so, can we develop accurate treatments that coordinate cytosolic DNA sensing and promote their clinical application.

## Pending issues

### The expression of cytosolic DNA sensors in various cell types and in different physical conditions

What is the specific mechanism underlying the selective expression of different types of cytosolic DNA sensors in various cell types upon physical or pathological stimulations? Does the same cytosolic DNA sensor exhibit preferential biological effects under different circumstances? With the comparison among the pathological features of different neurological diseases, we can illustrate the precise patterns of expression and effects of various kinds of cytosolic DNA sensors. This can enable us to identify the primary sensor involved in specific brain disorders, leading to the development of targeted treatments.

### The interaction among different kinds of cytosolic DNA sensors

Various types of cytosolic DNA sensors may co-exist in the same cell. As they all share the capacity to bind cytosolic DNA and activate innate immune response, they are assumed to be competitive or in cytosolic DNA sensing and downstream pathways. But cytosolic DNA sensors also cooperate with each other to enhance their biological influence. Revealing interaction networks among cytosolic DNA sensors will help us further understand their roles in different kinds of diseases.

### The evolution of cytosolic DNA sensors

Cytosolic DNA sensors differ in genetic loci and sequences, molecular structures, binding modes, and distribution, implicating they may have distinct and remote origins. How did these diverse molecules develop similar functions and biological effects, especially IFN-I production? Their evolution may provide hints to understand the relationship between cytosolic DNA sensors and neurodegenerative diseases.

### New therapies and treatments that target cytosolic DNA sensors in neurodegenerative diseases

Cytosolic DNA sensors have a major role in CNS diseases, and interference in the sensors or their key downstream regulators has shown some improvement of CNS diseases. But whether the regulation of cytosolic DNA sensors is sufficient to cure neurodegenerative diseases needs further research, and relative therapies and treatments are still far from clinical use.

### For more information

https://www.nature.com/subjects/dna-damage-and-repair/nature GLIAseq.com: https://gliaseqrev.ue.r.appspot.com Database of gene expression in adult mouse brain and lung vascular and perivascular cells: https://betsholtzlab.org/VascularSingleCells/database.html Azimuth: https://azimuth.hubmapconsortium.org/ Human_BBB: https://twc-stanford.shinyapps.io/human_bbb/ Tabula Muris: https://tabula-muris.ds.czbiohub.org/.

## Glossary

Innate immune response: The first line of defense against pathogenic invasion relying on physical barriers as well as cellular and molecular components. It is a rapid and non-specific response that is present from birth and does not target specific pathogens. It also activates adaptive immune response by antigen presentation.
Cyclic dinucleotides (CDNs): A type of small molecule composed of two nucleotides where their phosphate groups interlink to each other’s ribose via phosphodiester bonds to form a cyclic structure.
Mild cognitive impairment (MCI): The stage with noticeable loss of brain functions but no severe damage to self-care abilities, often regarded as the early hallmark of dementia.
Autophagy: A cell-autonomous degradation of unnecessary or detrimental molecules and organelles via the lysosome.
Inflammasome: A group of protein complexes that assemble under various stimuli to conduct innate immune response such as pro-inflammatory cytokine production and programmed cell death.
Interferon-stimulated genes (ISGs): A group of genes that undergo significant upregulation of expression in response to stimulation by interferon.
DNA double-strand break (DSB): Breakage of phosphodiester bonds in both strands of double-stranded DNA.

## References

[CR1] Abe T, Marutani Y, Shoji I (2019). Cytosolic DNA‐sensing immune response and viral infection. Microbiol Immunol.

[CR2] Albrecht M, Choubey D, Lengauer T (2005). The HIN domain of IFI-200 proteins consists of two OB folds. Biochem Biophys Res Commun.

[CR3] Almine JF, O’Hare CA, Dunphy G, Haga IR, Naik RJ, Atrih A, Connolly DJ, Taylor J, Kelsall IR, Bowie AG (2017). IFI16 and cGAS cooperate in the activation of STING during DNA sensing in human keratinocytes. Nat Commun.

[CR4] Andrisani O, Liu Q, Kehn P, Leitner WW, Moon K, Vazquez-Maldonado N, Fingerman I, Gale M (2022) Biological functions of DEAD/DEAH-box RNA helicases in health and disease. Nature Publishing Group. 10.1038/s41590-022-01149-7 PMC10259094 35194205

[CR5] Antiochos B, Trejo-Zambrano D, Fenaroli P, Rosenberg A, Baer A, Garg A, Sohn J, Li J, Petri M, Goldman DW (2022). The DNA sensors AIM2 and IFI16 are SLE autoantigens that bind neutrophil extracellular traps. ELife.

[CR6] Antonia RJ, Castillo J, Herring LE, Serafin DS, Liu P, Graves LM, Baldwin AS, Hagan RS (2019). TBK1 limits mTORC1 by promoting phosphorylation of raptor Ser877. Sci Rep.

[CR7] Balka KR, Louis C, Saunders TL, Smith AM, Calleja DJ, D’Silva DB, Moghaddas F, Tailler M, Lawlor KE, Zhan Y (2020). TBK1 and IKKε act redundantly to mediate STING-induced NF-κB responses in myeloid cells. Cell Rep.

[CR8] Barclay WE, Aggarwal N, Deerhake ME, Inoue M, Nonaka T, Nozaki K, Luzum NA, Miao EA, Shinohara ML (2022). The AIM2 inflammasome is activated in astrocytes during the late phase of EAE. JCI Insight.

[CR9] Bender A, Desplats P, Spencer B, Rockenstein E, Adame A, Elstner M, Laub C, Mueller S, Koob AO, Mante M (2013). TOM40 mediates mitochondrial dysfunction induced by α-synuclein accumulation in Parkinson’s disease. PLoS ONE.

[CR10] Bender A, Krishnan KJ, Morris CM, Taylor GA, Reeve AK, Perry RH, Jaros E, Hersheson JS, Betts J, Klopstock T (2006). High levels of mitochondrial DNA deletions in substantia nigra neurons in aging and Parkinson disease. Nat Genet.

[CR11] Bodur C, Kazyken D, Huang K, Ekim Ustunel B, Siroky KA, Tooley AS, Gonzalez IE, Foley DH, Acosta‐Jaquez HA, Barnes TM (2018). The IKK‐related kinase TBK1 activates mTORC1 directly in response to growth factors and innate immune agonists. EMBO J.

[CR12] Bolognesi C, Lando C, Forni A, Landini E, Scarpato R, Migliore L, Bonassi S (1999). Chromosomal damage and ageing: effect on micronuclei frequency in peripheral blood lymphocytes. Age Ageing.

[CR13] Bosso M, Kirchhoff F (2020). Emerging role of PYHIN proteins as antiviral restriction factors. Viruses.

[CR14] Brasnjevic I, Hof PR, Steinbusch HW, Schmitz C (2008). Accumulation of nuclear DNA damage or neuron loss: molecular basis for a new approach to understanding selective neuronal vulnerability in neurodegenerative diseases. DNA Repair.

[CR15] Braun S, Humphreys C, Fraser E, Brancale A, Bochtler M, Dale TC (2011). Amyloid-associated nucleic acid hybridisation. Plos One.

[CR16] Breijyeh Z, Karaman R (2020). Comprehensive review on Alzheimer’s disease: causes and treatment. Molecules.

[CR17] Bulloch K, Miller MM, Gal‐Toth J, Milner TA, Gottfried‐Blackmore A, Waters EM, Kaunzner UW, Liu K, Lindquist R, Nussenzweig MC (2008). CD11c/EYFP transgene illuminates a discrete network of dendritic cells within the embryonic, neonatal, adult, and injured mouse brain. J Comp Neurol.

[CR18] Burdette DL, Monroe KM, Sotelo-Troha K, Iwig JS, Eckert B, Hyodo M, Hayakawa Y, Vance RE (2011). STING is a direct innate immune sensor of cyclic di-GMP. Nature.

[CR19] Burdette DL, Vance RE (2013). STING and the innate immune response to nucleic acids in the cytosol. Nat Immunol.

[CR20] Cadena C, Hur S (2019). Filament-like assemblies of intracellular nucleic acid sensors: commonalities and differences. Mol Cell.

[CR21] Cai H, Holleufer A, Simonsen B, Schneider J, Lemoine A, Gad HH, Huang J, Huang J, Chen D, Peng T (2020). 2′ 3′-cGAMP triggers a STING-and NF-κB–dependent broad antiviral response in Drosophila. Sci Signal.

[CR22] Cai H, Imler J-L (2021). cGAS-STING: insight on the evolution of a primordial antiviral signaling cassette. Fac Rev.

[CR23] Cai H, Yan L, Liu N, Xu M, Cai H (2020). IFI16 promotes cervical cancer progression by upregulating PD-L1 in immunomicroenvironment through STING-TBK1-NF-kB pathway.. Biomed Pharmacother.

[CR24] Cao L-L, Guan P-P, Zhang S-Q, Yang Y, Huang X-S, Wang P (2021). Downregulating expression of OPTN elevates neuroinflammation via AIM2 inflammasome-and RIPK1-activating mechanisms in APP/PS1 transgenic mice. J Neuroinflammation.

[CR25] Chen H, Sun H, You F, Sun W, Zhou X, Chen L, Yang J, Wang Y, Tang H, Guan Y (2011). Activation of STAT6 by STING is critical for antiviral innate immunity. Cell.

[CR26] Chen J, Shu S, Chen Y, Liu Z, Yu L, Yang L, Xu Y, Zhang M (2019). AIM2 deletion promotes neuroplasticity and spatial memory of mice. Brain Res Bull.

[CR27] Chou W-C, Guo Z, Guo H, Chen L, Zhang G, Liang K, Xie L, Tan X, Gibson SA, Rampanelli E (2021). AIM2 in regulatory T cells restrains autoimmune diseases. Nature.

[CR28] Chu LH, Gangopadhyay A, Dorfleutner A, Stehlik C (2015). An updated view on the structure and function of PYRIN domains. Apoptosis.

[CR29] Civril F, Deimling T, de Oliveira Mann CC, Ablasser A, Moldt M, Witte G, Hornung V, Hopfner K-P (2013). Structural mechanism of cytosolic DNA sensing by cGAS. Nature.

[CR30] Constantinescu CS, Farooqi N, O’Brien K, Gran B (2011). Experimental autoimmune encephalomyelitis (EAE) as a model for multiple sclerosis (MS). Br J Pharmacol.

[CR31] Coppedè F, Migliore L (2015). DNA damage in neurodegenerative diseases. Mutat Res.

[CR32] Cox DJ, Field RH, Williams DG, Baran M, Bowie AG, Cunningham C, Dunne A (2015). DNA sensors are expressed in astrocytes and microglia in vitro and are upregulated during gliosis in neurodegenerative disease. Glia.

[CR33] Cree BA, Arnold DL, Chataway J, Chitnis T, Fox RJ, Ramajo AP, Murphy N, Lassmann H (2021). Secondary progressive multiple sclerosis: new insights. Neurology.

[CR34] Cridland JA, Curley EZ, Wykes MN, Schroder K, Sweet MJ, Roberts TL, Ragan MA, Kassahn KS, Stacey KJ (2012). The mammalian PYHIN gene family: phylogeny, evolution and expression. BMC Evol Biol.

[CR35] Crow YJ, Hayward BE, Parmar R, Robins P, Leitch A, Ali M, Black DN, Van Bokhoven H, Brunner HG, Hamel BC (2006). Mutations in the gene encoding the 3′-5′ DNA exonuclease TREX1 cause Aicardi-Goutieres syndrome at the AGS1 locus. Nat Genet.

[CR36] Cunha LD, Silva AL, Ribeiro JM, Mascarenhas DP, Quirino GF, Santos LL, Flavell RA, Zamboni DS (2017). AIM2 engages active but unprocessed caspase-1 to induce noncanonical activation of the NLRP3 inflammasome. Cell Rep.

[CR37] Daniels BP, Kofman SB, Smith JR, Norris GT, Snyder AG, Kolb JP, Gao X, Locasale JW, Martinez J, Gale M (2019). The nucleotide sensor ZBP1 and kinase RIPK3 induce the enzyme IRG1 to promote an antiviral metabolic state in neurons. Immunity.

[CR38] Dawson MJ, Trapani JA (1995). IFI 16 gene encodes a nuclear protein whose expression is induced by interferons in human myeloid leukaemia cell lines. J Cell Biochem.

[CR39] Deb P, Dai J, Singh S, Kalyoussef E, Fitzgerald-Bocarsly P (2020). Triggering of the cGAS–STING pathway in human plasmacytoid dendritic cells inhibits TLR9-mediated IFN production. J Immunol.

[CR40] Deigendesch N, Koch-Nolte F, Rothenburg S (2006). ZBP1 subcellular localization and association with stress granules is controlled by its Z-DNA binding domains. Nucleic Acids Res.

[CR41] Dell’Oste V, Gatti D, Gugliesi F, De Andrea M, Bawadekar M, Lo Cigno I, Biolatti M, Vallino M, Marschall M, Gariglio M (2014). Innate nuclear sensor IFI16 translocates into the cytoplasm during the early stage of in vitro human cytomegalovirus infection and is entrapped in the egressing virions during the late stage. J Virol.

[CR42] Deng L, Liang H, Xu M, Yang X, Burnette B, Arina A, Li X-D, Mauceri H, Beckett M, Darga T (2014). STING-dependent cytosolic DNA sensing promotes radiation-induced type I interferon-dependent antitumor immunity in immunogenic tumors. Immunity.

[CR43] de Rivero Vaccari JP, Brand FJ, Sedaghat C, Mash DC, Dietrich WD, Keane RW (2014). RIG-1 receptor expression in the pathology of Alzheimer’s disease. J Neuroinflammation.

[CR44] Ding R, Li H, Liu Y, Ou W, Zhang X, Chai H, Huang X, Yang W, Wang Q (2022). Activating cGAS–STING axis contributes to neuroinflammation in CVST mouse model and induces inflammasome activation and microglia pyroptosis. J Neuroinflammation.

[CR45] Domizio JD, Gulen MF, Saidoune F, Thacker VV, Yatim A, Sharma K, Nass T, Guenova E, Schaller M, Conrad C (2022). The cGAS–STING pathway drives type I IFN immunopathology in COVID-19. Nature.

[CR46] Eisenberg E, Levanon EY (2018). A-to-I RNA editing—immune protector and transcriptome diversifier. Nat Rev Genet.

[CR47] Ejlerskov P, Hultberg JG, Wang J, Carlsson R, Ambjørn M, Kuss M, Liu Y, Porcu G, Kolkova K, Rundsten CF (2015). Lack of neuronal IFN-β-IFNAR causes Lewy body-and Parkinson’s disease-like dementia. Cell.

[CR48] Erdal E, Haider S, Rehwinkel J, Harris AL, McHugh PJ (2017). A prosurvival DNA damage-induced cytoplasmic interferon response is mediated by end resection factors and is limited by Trex1.. Genes Dev.

[CR49] Ergun SL, Fernandez D, Weiss TM, Li L (2019). STING polymer structure reveals mechanisms for activation, hyperactivation, and inhibition. Cell.

[CR50] Fan X, Jiang J, Zhao D, Chen F, Ma H, Smith P, Unterholzner L, Xiao TS, Jin T (2021). Structural mechanism of DNA recognition by the p204 HIN domain. Nucleic Acids Res.

[CR51] Fenech M, Kirsch-Volders M, Natarajan A, Surralles J, Crott J, Parry J, Norppa H, Eastmond D, Tucker JD, Thomas P (2011). Molecular mechanisms of micronucleus, nucleoplasmic bridge and nuclear bud formation in mammalian and human cells. Mutagenesis.

[CR52] Fujiuchi N, Aglipay JA, Ohtsuka T, Maehara N, Sahin F, Su GH, Lee SW, Ouchi T (2004). Requirement of IFI16 for the maximal activation of p53 induced by ionizing radiation. J Biol Chem.

[CR53] Gao J, Peng S, Shan X, Deng G, Shen L, Sun J, Jiang C, Yang X, Chang Z, Sun X (2019). Inhibition of AIM2 inflammasome-mediated pyroptosis by Andrographolide contributes to amelioration of radiation-induced lung inflammation and fibrosis. Cell Death Dis.

[CR54] Giordano AMS, Luciani M, Gatto F, Abou Alezz M, Beghè C, Della Volpe L, Migliara A, Valsoni S, Genua M, Dzieciatkowska M (2022). DNA damage contributes to neurotoxic inflammation in Aicardi-Goutières syndrome astrocytes. J Exp Med.

[CR55] Goto A, Okado K, Martins N, Cai H, Barbier V, Lamiable O, Troxler L, Santiago E, Kuhn L, Paik D (2018). The kinase IKKβ regulates a STING-and NF-κB-dependent antiviral response pathway in Drosophila. Immunity.

[CR56] Gui X, Yang H, Li T, Tan X, Shi P, Li M, Du F, Chen ZJ (2019). Autophagy induction via STING trafficking is a primordial function of the cGAS pathway. Nature.

[CR57] Gulen MF, Samson N, Keller A, Schwabenland M, Liu C, Glück S, Thacker VV, Favre L, Mangeat B, Kroese LJ (2023) cGAS–STING drives ageing-related inflammation and neurodegeneration. Nature 620:374–380 10.1038/s41586-023-06373-1 PMC10412454 37532932

[CR58] Guo J, Wang J, Fan J, Zhang Y, Dong W, Chen CP (2019). Distinct dynamic and conformational features of human STING in response to 2′ 3′‐cGAMP and c‐di‐GMP. Chembiochem.

[CR59] Guo Y, Gu R, Gan D, Hu F, Li G, Xu G (2020). Mitochondrial DNA drives noncanonical inflammation activation via cGAS–STING signaling pathway in retinal microvascular endothelial cells. Cell Commun Signal.

[CR60] Ha SC, Kim D, Hwang H-Y, Rich A, Kim Y-G, Kim KK (2008). The crystal structure of the second Z-DNA binding domain of human DAI (ZBP1) in complex with Z-DNA reveals an unusual binding mode to Z-DNA. Proc Natl Acad Sci USA.

[CR61] Ha SC, Van Quyen D, Hwang H-Y, Oh D-B, Brown IIBA, Lee SM, Park H-J, Ahn J-H, Kim KK, Kim Y-G (2006). Biochemical characterization and preliminary X-ray crystallographic study of the domains of human ZBP1 bound to left-handed Z-DNA. Biochim Biophys Acta.

[CR62] Hasan M, Gonugunta VK, Dobbs N, Ali A, Palchik G, Calvaruso MA, DeBerardinis RJ, Yan N (2017). Chronic innate immune activation of TBK1 suppresses mTORC1 activity and dysregulates cellular metabolism. Proc Natl Acad Sci USA.

[CR63] He L, Vanlandewijck M, Mäe MA, Andrae J, Ando K, Del Gaudio F, Nahar K, Lebouvier T, Laviña B, Gouveia L (2018). Single-cell RNA sequencing of mouse brain and lung vascular and vessel-associated cell types. Sci Data.

[CR64] Herzner A-M, Hagmann CA, Goldeck M, Wolter S, Kübler K, Wittmann S, Gramberg T, Andreeva L, Hopfner K-P, Mertens C (2015). Sequence-specific activation of the DNA sensor cGAS by Y-form DNA structures as found in primary HIV-1 cDNA. Nat Immunol.

[CR65] Hinkle JT, Patel J, Panicker N, Karuppagounder SS, Biswas D, Belingon B, Chen R, Brahmachari S, Pletnikova O, Troncoso JC (2022). STING mediates neurodegeneration and neuroinflammation in nigrostriatal α-synucleinopathy. Proc Natl Acad Sci USA.

[CR66] Hobson EV, McDermott CJ (2016). Supportive and symptomatic management of amyotrophic lateral sclerosis. Nat Rev Neurol.

[CR67] Hojati Z, Kay M, Dehghanian F (2016) Mechanism of action of interferon beta in treatment of multiple sclerosis. In: Multiple sclerosis. Elsevier, pp 365–392

[CR68] Holleufer A, Winther KG, Gad HH, Ai X, Chen Y, Li L, Wei Z, Deng H, Liu J, Frederiksen NA (2021). Two cGAS-like receptors induce antiviral immunity in Drosophila. Nature.

[CR69] Hong Z, Mei J, Li C, Bai G, Maimaiti M, Hu H, Yu W, Sun L, Zhang L, Cheng D (2021). STING inhibitors target the cyclic dinucleotide binding pocket. Proc Natl Acad Sci USA.

[CR70] Hornung V, Hartmann R, Ablasser A, Hopfner K-P (2014). OAS proteins and cGAS: unifying concepts in sensing and responding to cytosolic nucleic acids. Nat Rev Immunol.

[CR71] Hou Y, Wei Y, Lautrup S, Yang B, Wang Y, Cordonnier S, Mattson MP, Croteau DL, Bohr VA (2021). NAD+ supplementation reduces neuroinflammation and cell senescence in a transgenic mouse model of Alzheimer’s disease via cGAS–STING. Proc Natl Acad Sci USA.

[CR72] Howard TR, Lum KK, Kennedy MA, Cristea IM (2022) The nuclear DNA sensor IFI16 indiscriminately binds to and diminishes accessibility of the HSV-1 genome to suppress infection. Msystems 7:e00198-00122 10.1128/msystems.00198-22 PMC9239196 35575489

[CR73] Hu B, Jin C, Li H-B, Tong J, Ouyang X, Cetinbas NM, Zhu S, Strowig T, Lam FC, Zhao C (2016). The DNA-sensing AIM2 inflammasome controls radiation-induced cell death and tissue injury. Science.

[CR74] Huang LS, Hong Z, Wu W, Xiong S, Zhong M, Gao X, Rehman J, Malik AB (2020). mtDNA activates cGAS signaling and suppresses the YAP-mediated endothelial cell proliferation program to promote inflammatory injury. Immunity.

[CR75] Hur S (2019). Double-stranded RNA sensors and modulators in innate immunity. Ann Rev Immunol.

[CR76] Hurst TP, Aswad A, Karamitros T, Katzourakis A, Smith AL, Magiorkinis G (2019). Interferon-inducible protein 16 (IFI16) has a broad-spectrum binding ability against ssDNA targets: an evolutionary hypothesis for antiretroviral checkpoint. Front Microbiol.

[CR77] Iannucci A, Caneparo V, Raviola S, Debernardi I, Colangelo D, Miggiano R, Griffante G, Landolfo S, Gariglio M, De Andrea M (2020). Toll-like receptor 4-mediated inflammation triggered by extracellular IFI16 is enhanced by lipopolysaccharide binding. PLoS Pathog.

[CR78] Jankovic J, Tan EK (2020). Parkinson’s disease: etiopathogenesis and treatment. J Neurol Neurosurg Psychiatry.

[CR79] Jeffries A, Marriott I (2017) Human astrocytes and microglia-like cells express cGAS-STING viral sensing components. J Immunol 198(1_Supplement):129.6 10.1016/j.neulet.2017.08.039 PMC5645252 28830822

[CR80] Jeffries AM, Suptela AJ, Marriott I (2022). Z-DNA binding protein 1 mediates necroptotic and apoptotic cell death pathways in murine astrocytes following herpes simplex virus-1 infection. J Neuroinflammation.

[CR81] Jeffries AM, Truman AW, Marriott I (2020). The intracellular DNA sensors cGAS and IFI16 do not mediate effective antiviral immune responses to HSV-1 in human microglial cells. J Neurovirol.

[CR82] Jiang H, Swacha P, Gekara NO (2021). Nuclear AIM2‐Like receptors drive genotoxic tissue injury by inhibiting DNA repair. Adv Sci.

[CR83] Jiang H, Xue X, Panda S, Kawale A, Hooy RM, Liang F, Sohn J, Sung P, Gekara NO (2019). Chromatin‐bound cGAS is an inhibitor of DNA repair and hence accelerates genome destabilization and cell death. EMBO J.

[CR84] Jiang Y, Zhu Y, Qiu W, Liu Y-J, Cheng G, Liu Z-J, Ouyang S (2017). Structural and functional analyses of human DDX41 DEAD domain. Protein Cell.

[CR85] Jiao H, Wachsmuth L, Kumari S, Schwarzer R, Lin J, Eren RO, Fisher A, Lane R, Young GR, Kassiotis G (2020). Z-nucleic-acid sensing triggers ZBP1-dependent necroptosis and inflammation. Nature.

[CR86] Jin J, Xiao Y, Chang J-H, Yu J, Hu H, Starr R, Brittain GC, Chang M, Cheng X, Sun S-C (2012). The kinase TBK1 controls IgA class switching by negatively regulating noncanonical NF-κB signaling. Nat Immunol.

[CR87] Jin M, Shiwaku H, Tanaka H, Obita T, Ohuchi S, Yoshioka Y, Jin X, Kondo K, Fujita K, Homma H (2021). Tau activates microglia via the PQBP1-cGAS-STING pathway to promote brain inflammation. Nat Commun.

[CR88] Jin T, Perry A, Jiang J, Smith P, Curry JA, Unterholzner L, Jiang Z, Horvath G, Rathinam VA, Johnstone RW (2012). Structures of the HIN domain: DNA complexes reveal ligand binding and activation mechanisms of the AIM2 inflammasome and IFI16 receptor. Immunity.

[CR89] Johnson BM, Uchimura T, Gallovic MD, Thamilarasan M, Chou W-C, Gibson SA, Deng M, Tam JW, Batty CJ, Williams J (2021). STING agonist mitigates experimental autoimmune encephalomyelitis by stimulating type I IFN–dependent and–independent immune-regulatory pathways. J Immunol.

[CR90] Johnson KE, Bottero V, Flaherty S, Dutta S, Singh VV, Chandran B (2014). IFI16 restricts HSV-1 replication by accumulating on the hsv-1 genome, repressing HSV-1 gene expression, and directly or indirectly modulating histone modifications. PLoS Pathog.

[CR91] Jønsson K, Laustsen A, Krapp C, Skipper K, Thavachelvam K, Hotter D, Egedal J, Kjolby M, Mohammadi P, Prabakaran T (2017). IFI16 is required for DNA sensing in human macrophages by promoting production and function of cGAMP. Nat Commun.

[CR92] Ka N-L, Lim GY, Hwang S, Kim S-S, Lee M-O (2021). IFI16 inhibits DNA repair that potentiates type-I interferon-induced antitumor effects in triple negative breast cancer. Cell Rep.

[CR93] Kato K, Ahmad S, Zhu Z, Young JM, Mu X, Park S, Malik HS, Hur S (2021). Structural analysis of RIG-I-like receptors reveals ancient rules of engagement between diverse RNA helicases and TRIM ubiquitin ligases. Mol Cell.

[CR94] Kerur N, Veettil MV, Sharma-Walia N, Bottero V, Sadagopan S, Otageri P, Chandran B (2011). IFI16 acts as a nuclear pathogen sensor to induce the inflammasome in response to Kaposi Sarcoma-associated herpesvirus infection. Cell Host Microbe.

[CR95] Kiernan MC, Vucic S, Cheah BC, Turner MR, Eisen A, Hardiman O, Burrell JR, Zoing MC (2011). Amyotrophic lateral sclerosis. Lancet.

[CR96] Kim T, Pazhoor S, Bao M, Zhang Z, Hanabuchi S, Facchinetti V, Bover L, Plumas J, Chaperot L, Qin J (2010). Aspartate-glutamate-alanine-histidine box motif (DEAH)/RNA helicase A helicases sense microbial DNA in human plasmacytoid dendritic cells. Proc Natl Acad Sci USA.

[CR97] Kister I, Bacon TE, Chamot E, Salter AR, Cutter GR, Kalina JT, Herbert J (2013). Natural history of multiple sclerosis symptoms. Int J MS Care.

[CR98] Konno H, Konno K, Barber GN (2013). Cyclic dinucleotides trigger ULK1 (ATG1) phosphorylation of STING to prevent sustained innate immune signaling. Cell.

[CR99] Kranzusch PJ, Lee AS-Y, Berger JM, Doudna JA (2013). Structure of human cGAS reveals a conserved family of second-messenger enzymes in innate immunity. Cell Rep.

[CR100] Kuriakose T, Kanneganti T-D (2018). ZBP1: innate sensor regulating cell death and inflammation. Trends Immunol.

[CR240] Kuriakose T, Zheng M, Neale G, Kanneganti T-D (2018) IRF1 is a transcriptional regulator of ZBP1 promoting NLRP3 inflammasome activation and cell death during influenza virus infection. J Immunol 200(4):1489–1495 10.4049/jimmunol.1701538 PMC6483084 29321274

[CR101] Kwak JC, Ongusaha PP, Ouchi T, Lee SW (2003). IFI16 as a negative regulator in the regulation of p53 and p21Waf1. J Biol Chem.

[CR102] Lammert CR, Frost EL, Bellinger CE, Bolte AC, McKee CA, Hurt ME, Paysour MJ, Ennerfelt HE, Lukens JR (2020). AIM2 inflammasome surveillance of DNA damage shapes neurodevelopment. Nature.

[CR103] Lan YY, Heather JM, Eisenhaure T, Garris CS, Lieb D, Raychowdhury R, Hacohen N (2019). Extranuclear DNA accumulates in aged cells and contributes to senescence and inflammation. Aging Cell.

[CR104] Lassmann H, Van Horssen J, Mahad D (2012). Progressive multiple sclerosis: pathology and pathogenesis. Nat Rev Neurol.

[CR105] Lee K-G, Kim SS-Y, Kui L, Voon DC-C, Mauduit M, Bist P, Bi X, Pereira NA, Liu C, Sukumaran B (2015). Bruton’s tyrosine kinase phosphorylates DDX41 and activates its binding of dsDNA and STING to initiate type 1 interferon response. Cell Rep.

[CR106] Li D, Wu R, Guo W, Xie L, Qiao Z, Chen S, Zhu J, Huang C, Huang J, Chen B (2019). STING-mediated IFI16 degradation negatively controls type I interferon production. Cell Rep.

[CR107] Li F, Wang N, Zheng Y, Luo Y, Zhang Y (2021). cGAS-stimulator of interferon genes signaling in central nervous system disorders. Aging Dis.

[CR108] Li S, Qu L, Wang X, Kong L (2021). Novel insights into RIPK1 as a promising target for future Alzheimer’s disease treatment. Pharmacol Ther.

[CR109] Li X, Li X, Xie C, Cai S, Li M, Jin H, Wu S, Cui J, Liu H, Zhao Y (2022). cGAS guards against chromosome end-to-end fusions during mitosis and facilitates replicative senescence. Protein Cell.

[CR110] Liao JC, Lam R, Brazda V, Duan S, Ravichandran M, Ma J, Xiao T, Tempel W, Zuo X, Wang Y-X (2011). Interferon-inducible protein 16: insight into the interaction with tumor suppressor p53. Structure.

[CR111] Lin J, Kumari S, Kim C, Van T-M, Wachsmuth L, Polykratis A, Pasparakis M (2016). RIPK1 counteracts ZBP1-mediated necroptosis to inhibit inflammation. Nature.

[CR112] Lin X, Kapoor A, Gu Y, Chow MJ, Peng J, Zhao K, Tang D (2020). Contributions of DNA damage to Alzheimer’s disease. Int J Mol Sci.

[CR113] Liu C, Zhang Y (2011). Nucleic acid-mediated protein aggregation and assembly. Adv Protein Chem Struct Biol.

[CR114] Liu D, Wu H, Wang C, Li Y, Tian H, Siraj S, Sehgal SA, Wang X, Wang J, Shang Y (2019). STING directly activates autophagy to tune the innate immune response. Cell Death Differ.

[CR115] Liu H, Zhang H, Wu X, Ma D, Wu J, Wang L, Jiang Y, Fei Y, Zhu C, Tan R (2018). Nuclear cGAS suppresses DNA repair and promotes tumorigenesis. Nature.

[CR116] Liu T, Tang Q, Liu K, Xie W, Liu X, Wang H, Wang R-F, Cui J (2016). TRIM11 suppresses AIM2 inflammasome by degrading AIM2 via p62-dependent selective autophagy. Cell Rep.

[CR117] Liu Y, Gordesky-Gold B, Leney-Greene M, Weinbren NL, Tudor M, Cherry S (2018). Inflammation-induced, STING-dependent autophagy restricts Zika virus infection in the Drosophila brain. Cell Host Microbe.

[CR118] López-Otín C, Blasco MA, Partridge L, Serrano M, Kroemer G (2023). Hallmarks of aging: an expanding universe. Cell.

[CR119] Lu F, Selak M, O’Connor J, Croul S, Lorenzana C, Butunoi C, Kalman B (2000). Oxidative damage to mitochondrial DNA and activity of mitochondrial enzymes in chronic active lesions of multiple sclerosis. J Neurol Sci.

[CR120] Luecke S, Holleufer A, Christensen MH, Jønsson KL, Boni GA, Sørensen LK, Johannsen M, Jakobsen MR, Hartmann R, Paludan SR (2017). cGAS is activated by DNA in a length‐dependent manner. EMBO Rep.

[CR121] Luo W, Wang Y, Zhang L, Ren P, Zhang C, Li Y, Azares AR, Zhang M, Guo J, Ghaghada KB (2020). Critical role of cytosolic DNA and its sensing adaptor STING in aortic degeneration, dissection, and rupture. Circulation.

[CR122] Luo X, Li H, Ma L, Zhou J, Guo X, Woo S-L, Pei Y, Knight LR, Deveau M, Chen Y (2018). Expression of STING is increased in liver tissues from patients with NAFLD and promotes macrophage-mediated hepatic inflammation and fibrosis in mice. Gastroenterology.

[CR123] Ma C, Li S, Hu Y, Ma Y, Wu Y, Wu C, Liu X, Wang B, Hu G, Zhou J (2021). AIM2 controls microglial inflammation to prevent experimental autoimmune encephalomyelitis. J Exp Med.

[CR124] Ma F, Li B, Liu S-y, Iyer SS, Yu Y, Wu A, Cheng G (2015). Positive feedback regulation of type I IFN production by the IFN-inducible DNA sensor cGAS. J Immunol.

[CR125] Mackenzie KJ, Carroll P, Martin C-A, Murina O, Fluteau A, Simpson DJ, Olova N, Sutcliffe H, Rainger JK, Leitch A (2017). cGAS surveillance of micronuclei links genome instability to innate immunity. Nature.

[CR126] Main BS, Zhang M, Brody KM, Ayton S, Frugier T, Steer D, Finkelstein D, Crack PJ, Taylor JM (2016). Type‐1 interferons contribute to the neuroinflammatory response and disease progression of the MPTP mouse model of Parkinson’s disease. Glia.

[CR127] Main BS, Zhang M, Brody KM, Kirby FJ, Crack PJ, Taylor JM (2017). Type‐I interferons mediate the neuroinflammatory response and neurotoxicity induced by rotenone. J Neurochem.

[CR128] Mankan AK, Schmidt T, Chauhan D, Goldeck M, Höning K, Gaidt M, Kubarenko AV, Andreeva L, Hopfner KP, Hornung V (2014). Cytosolic RNA: DNA hybrids activate the cGAS–STING axis. EMBO J.

[CR129] Mao P, Reddy PH (2011). Aging and amyloid beta-induced oxidative DNA damage and mitochondrial dysfunction in Alzheimer’s disease: implications for early intervention and therapeutics. Biochim Biophys Acta.

[CR130] Marcelo A, Koppenol R, de Almeida LP, Matos CA, Nóbrega C (2021). Stress granules, RNA-binding proteins and polyglutamine diseases: too much aggregation?. Cell Death Dis.

[CR131] Margolis SR, Wilson SC, Vance RE (2017). Evolutionary origins of cGAS-STING signaling. Trends Immunol.

[CR132] Mathys H, Adaikkan C, Gao F, Young JZ, Manet E, Hemberg M, De Jager PL, Ransohoff RM, Regev A, Tsai L-H (2017). Temporal tracking of microglia activation in neurodegeneration at single-cell resolution. Cell Rep.

[CR133] Matsui H, Ito J, Matsui N, Uechi T, Onodera O, Kakita A (2021). Cytosolic dsDNA of mitochondrial origin induces cytotoxicity and neurodegeneration in cellular and zebrafish models of Parkinson’s disease. Nat Commun.

[CR134] Matyszewski M, Morrone SR, Sohn J (2018). Digital signaling network drives the assembly of the AIM2-ASC inflammasome. Proc Natl Acad Sci USA.

[CR135] McCauley ME, O’Rourke JG, Yáñez A, Markman JL, Ho R, Wang X, Chen S, Lall D, Jin M, Muhammad A (2020). C9orf72 in myeloid cells suppresses STING-induced inflammation. Nature.

[CR136] McCubrey J, May WS, Duronio V, Mufson A (2000). Serine/threonine phosphorylation in cytokine signal transduction. Leukemia.

[CR137] McKenzie BA, Mamik MK, Saito LB, Boghozian R, Monaco MC, Major EO, Lu J-Q, Branton WG, Power C (2018). Caspase-1 inhibition prevents glial inflammasome activation and pyroptosis in models of multiple sclerosis. Proc Natl Acad Sci USA.

[CR138] Migliore L, Coppedè F, Fenech M, Thomas P (2011). Association of micronucleus frequency with neurodegenerative diseases. Mutagenesis.

[CR139] Milstead RA, Link CD, Xu Z, Hoeffer CA (2023). TDP-43 knockdown in mouse model of ALS leads to dsRNA deposition, gliosis, and neurodegeneration in the spinal cord. Cereb Cortex.

[CR140] Mittal M (2021) Cyclic GMP-AMP synthase regulates the development of experimental autoimmune encephalomyelitis. The University of Iowa.

[CR141] Mohamed E, Lemos H, Huang L, Ou R, Pacholczyk G, Hayakawa Y, Munn D, Mellor A (2015) DNA sensing via STING regulates autoimmunity and tumor immunity. (IRM5P. 648). J Immunol 194(1_Supplement):59.13

[CR142] Möller M, Wasel J, Schmetzer J, Weiß U, Meissner M, Schiffmann S, Weigert A, Möser CV, Niederberger E (2020). The specific IKKε/TBK1 inhibitor Amlexanox suppresses human melanoma by the inhibition of autophagy, NF-κB and MAP kinase pathways. Int J Mol Sci.

[CR143] Moore Z, Mobilio F, Walker FR, Taylor JM, Crack PJ (2020). Abrogation of type-I interferon signalling alters the microglial response to Aβ1–42. Sci Rep.

[CR144] Morand EF, Furie R, Tanaka Y, Bruce IN, Askanase AD, Richez C, Bae S-C, Brohawn PZ, Pineda L, Berglind A (2020). Trial of anifrolumab in active systemic lupus erythematosus. N Engl J Med.

[CR145] Morrone SR, Matyszewski M, Yu X, Delannoy M, Egelman EH, Sohn J (2015). Assembly-driven activation of the AIM2 foreign-dsDNA sensor provides a polymerization template for downstream ASC. Nat Commun.

[CR146] Mosler T, Conte F, Longo GM, Mikicic I, Kreim N, Möckel MM, Petrosino G, Flach J, Barau J, Luke B (2021). R-loop proximity proteomics identifies a role of DDX41 in transcription-associated genomic instability. Nat Commun.

[CR147] Motani K, Kosako H (2020). BioID screening of biotinylation sites using the avidin-like protein Tamavidin 2-REV identifies global interactors of stimulator of interferon genes (STING). J Biol Chem.

[CR148] Muendlein HI, Connolly WM, Magri Z, Jetton D, Smirnova I, Degterev A, Balachandran S, Poltorak A (2022). ZBP1 promotes inflammatory responses downstream of TLR3/TLR4 via timely delivery of RIPK1 to TRIF. Proc Natl Acad Sci USA.

[CR149] Muendlein HI, Connolly WM, Magri Z, Smirnova I, Ilyukha V, Gautam A, Degterev A, Poltorak A (2021). ZBP1 promotes LPS-induced cell death and IL-1β release via RHIM-mediated interactions with RIPK1. Nat Commun.

[CR150] Nakanishi K, Akira S (2000). NF‐κB activation through IKK‐i‐dependent I‐TRAF/TANK phosphorylation. Genes Cells.

[CR151] Nazmi A, Field RH, Griffin EW, Haugh O, Hennessy E, Cox D, Reis R, Tortorelli L, Murray CL, Lopez‐Rodriguez AB (2019). Chronic neurodegeneration induces type I interferon synthesis via STING, shaping microglial phenotype and accelerating disease progression. Glia.

[CR152] Ni X, Ru H, Ma F, Zhao L, Shaw N, Feng Y, Ding W, Gong W, Wang Q, Ouyang S (2016). New insights into the structural basis of DNA recognition by HINa and HINb domains of IFI16. J Mol Cell Biol.

[CR153] Nichols E, Steinmetz JD, Vollset SE, Fukutaki K, Chalek J, Abd-Allah F, Abdoli A, Abualhasan A, Abu-Gharbieh E, Akram TT (2022). Estimation of the global prevalence of dementia in 2019 and forecasted prevalence in 2050: an analysis for the Global Burden of Disease Study 2019. Lancet Public Health.

[CR154] Nourse JB, Russell SN, Moniz NA, Peter K, Seyfarth LM, Scott M, Park H-A, Caldwell KA, Caldwell GA (2023). Integrated regulation of dopaminergic and epigenetic effectors of neuroprotection in Parkinson’s disease models. Proc Natl Acad Sci USA.

[CR155] Ochoa E, Ramirez P, Gonzalez E, De Mange J, Ray WJ, Bieniek KF, Frost B (2023). Pathogenic tau–induced transposable element–derived dsRNA drives neuroinflammation. Sci Adv.

[CR156] Ogawa E, Mukai K, Saito K, Arai H, Taguchi T (2018). The binding of TBK1 to STING requires exocytic membrane traffic from the ER. Biochem Biophys Res Commun.

[CR157] Omura H, Oikawa D, Nakane T, Kato M, Ishii R, Ishitani R, Tokunaga F, Nureki O (2016). Structural and Functional Analysis of DDX41: a bispecific immune receptor for DNA and cyclic dinucleotide. Sci Rep.

[CR158] Orzalli MH, Broekema NM, Diner BA, Hancks DC, Elde NC, Cristea IM, Knipe DM (2015). cGAS-mediated stabilization of IFI16 promotes innate signaling during herpes simplex virus infection. Proc Natl Acad Sci USA.

[CR159] Ouchi M, Ouchi T (2008). Role of IFI16 in DNA damage and checkpoint. Front Biosc.

[CR160] Ouyang S, Song X, Wang Y, Ru H, Shaw N, Jiang Y, Niu F, Zhu Y, Qiu W, Parvatiyar K (2012). Structural analysis of the STING adaptor protein reveals a hydrophobic dimer interface and mode of cyclic di-GMP binding. Immunity.

[CR161] Pao P-C, Patnaik D, Watson LA, Gao F, Pan L, Wang J, Adaikkan C, Penney J, Cam HP, Huang W-C (2020). HDAC1 modulates OGG1-initiated oxidative DNA damage repair in the aging brain and Alzheimer’s disease. Nat Commun.

[CR162] Paolicelli RC, Jawaid A, Henstridge CM, Valeri A, Merlini M, Robinson JL, Lee EB, Rose J, Appel S, Lee VM-Y (2017). TDP-43 depletion in microglia promotes amyloid clearance but also induces synapse loss. Neuron.

[CR163] Parvatiyar K, Zhang Z, Teles RM, Ouyang S, Jiang Y, Iyer SS, Zaver SA, Schenk M, Zeng S, Zhong W (2013) DDX41 recognizes bacterial secondary messengers cyclic di-GMP and cyclic di-AMP to activate a type I interferon immune response. In: Journal of Immunology. AMER ASSOC IMMUNOLOGISTS 9650 ROCKVILLE PIKE, BETHESDA, MD 20814, USA 10.1038/ni.2460 PMC3501571 23142775

[CR164] Pham PT, Fukuda D, Nishimoto S, Kim-Kaneyama J-R, Lei X-F, Takahashi Y, Sato T, Tanaka K, Suto K, Kawabata Y (2021). STING, a cytosolic DNA sensor, plays a critical role in atherogenesis: a link between innate immunity and chronic inflammation caused by lifestyle-related diseases. Eur Heart J.

[CR165] Philipp J, Le Gleut R, Toerne C, Subedi P, Azimzadeh O, Atkinson MJ, Tapio S (2020). Radiation response of human cardiac endothelial cells reveals a central role of the cGAS-STING pathway in the development of inflammation. Proteomes.

[CR166] Pomerantz JL, Baltimore D (1999). NF-κB activation by a signaling complex containing TRAF2, TANK and TBK1, a novel IKK-related kinase. EMBO J.

[CR167] Prabakaran T, Bodda C, Krapp C, Zhang B, Christensen MH, Sun C, Reinert L, Cai Y, Jensen SB, Skouboe MK (2018). Attenuation of c GAS‐STING signaling is mediated by a p62/SQSTM 1‐dependent autophagy pathway activated by TBK1. EMBO J.

[CR168] Patterson C (2018) World alzheimer report 2018.

[CR169] Quan M-Y, Song X-J, Liu H-J, Deng X-H, Hou H-Q, Chen L-P, Ma T-Z, Han X, He X-X, Jia Z (2019). Amlexanox attenuates experimental autoimmune encephalomyelitis by inhibiting dendritic cell maturation and reprogramming effector and regulatory T cell responses. J Neuroinflammation.

[CR170] Radak M, Fallahi H (2023) Zbp1 gene: a modulator of multiple aging hallmarks as potential therapeutic target for age-related diseases. Biogerontology 24:831–844 10.1007/s10522-023-10039-w 37199888

[CR171] Rebsamen M, Heinz LX, Meylan E, Michallet MC, Schroder K, Hofmann K, Vazquez J, Benedict CA, Tschopp J (2009). DAI/ZBP1 recruits RIP1 and RIP3 through RIP homotypic interaction motifs to activate NF‐κB. EMBO Rep.

[CR172] Rehwinkel J, Gack MU (2020). RIG-I-like receptors: their regulation and roles in RNA sensing. Nat Rev Immunol.

[CR173] Reilly SM, Chiang S-H, Decker SJ, Chang L, Uhm M, Larsen MJ, Rubin JR, Mowers J, White NM, Hochberg I (2013). An inhibitor of the protein kinases TBK1 and IKK-ɛ improves obesity-related metabolic dysfunctions in mice. Nat Med.

[CR174] Reinert LS, Rashidi AS, Tran DN, Katzilieris-Petras G, Hvidt AK, Gohr M, Fruhwürth S, Bodda C, Thomsen MK, Vendelbo MH (2021). Brain immune cells undergo cGAS/STING-dependent apoptosis during herpes simplex virus type 1 infection to limit type I IFN production. J Clin Invest.

[CR175] Rexach JE, Polioudakis D, Yin A, Swarup V, Chang TS, Nguyen T, Sarkar A, Chen L, Huang J, Lin L-C (2020). Tau pathology drives dementia risk-associated gene networks toward chronic inflammatory states and immunosuppression. Cell Rep.

[CR176] Rice G, Ebers G (1998). Interferons in the treatment of multiple sclerosis: do they prevent the progression of the disease?. Arch Neurol.

[CR177] Rong Y, Zhang S, Nandi N, Wu Z, Li L, Liu Y, Wei Y, Zhao Y, Yuan W, Zhou C (2022). STING controls energy stress-induced autophagy and energy metabolism via STX17. J Cell Biol.

[CR178] Roy A, Ghosh A, Kumar B, Chandran B (2019). IFI16, a nuclear innate immune DNA sensor, mediates epigenetic silencing of herpesvirus genomes by its association with H3K9 methyltransferases SUV39H1 and GLP. Elife.

[CR179] Roy ER, Chiu G, Li S, Propson NE, Kanchi R, Wang B, Coarfa C, Zheng H, Cao W (2022). Concerted type I interferon signaling in microglia and neural cells promotes memory impairment associated with amyloid β plaques. Immunity.

[CR180] Roy ER, Wang B, Wan Y-w, Chiu G, Cole A, Yin Z, Propson NE, Xu Y, Jankowsky JL, Liu Z (2020). Type I interferon response drives neuroinflammation and synapse loss in Alzheimer disease. J Clin Invest.

[CR181] Rui W, Xiao H, Fan Y, Ma Z, Xiao M, Li S, Shi J (2021). Systemic inflammasome activation and pyroptosis associate with the progression of amnestic mild cognitive impairment and Alzheimer’s disease. J Neuroinflammation.

[CR182] Rui WJ, Li S, Yang L, Liu Y, Fan Y, Hu YC, Ma CM, Wang BW, Shi JP (2022). Microglial AIM2 alleviates antiviral‐related neuro‐inflammation in mouse models of Parkinson’s disease. Glia.

[CR183] Saada J, McAuley RJ, Marcatti M, Tang TZ, Motamedi M, Szczesny B (2022). Oxidative stress induces Z-DNA-binding protein 1–dependent activation of microglia via mtDNA released from retinal pigment epithelial cells. J Biol Chem.

[CR184] Saitoh T, Fujita N, Hayashi T, Takahara K, Satoh T, Lee H, Matsunaga K, Kageyama S, Omori H, Noda T (2009). Atg9a controls dsDNA-driven dynamic translocation of STING and the innate immune response. Proc Natl Acad Sci USA.

[CR185] Schaser AJ, Osterberg VR, Dent SE, Stackhouse TL, Wakeham CM, Boutros SW, Weston LJ, Owen N, Weissman TA, Luna E (2019). Alpha-synuclein is a DNA binding protein that modulates DNA repair with implications for Lewy body disorders. Sci Rep.

[CR186] Schwartz T, Behlke J, Lowenhaupt K, Heinemann U, Rich A (2001). Structure of the DLM-1–Z-DNA complex reveals a conserved family of Z-DNA-binding proteins. Nat Struct Biol.

[CR187] Shanbhag NM, Evans MD, Mao W, Nana AL, Seeley WW, Adame A, Rissman RA, Masliah E, Mucke L (2019). Early neuronal accumulation of DNA double strand breaks in Alzheimer’s disease. Acta Neuropathol Commun.

[CR188] Shen Y, Lam A, Ho S, Koo C, Le Bert N, Gasser S (2014) Cancer pathogenesis and DNA sensing. In: Biological DNA sensor. Elsevier, pp. 205–229

[CR189] Singh RS, Vidhyasagar V, Yang S, Arna AB, Yadav M, Aggarwal A, Aguilera AN, Shinriki S, Bhanumathy KK, Pandey K (2022). DDX41 is required for cGAS-STING activation against DNA virus infection. Cell Rep.

[CR190] Sladitschek-Martens HL, Guarnieri A, Brumana G, Zanconato F, Battilana G, Xiccato RL, Panciera T, Forcato M, Bicciato S, Guzzardo V (2022). YAP/TAZ activity in stromal cells prevents ageing by controlling cGAS–STING. Nature.

[CR191] Slavik KM, Morehouse BR, Ragucci AE, Zhou W, Ai X, Chen Y, Li L, Wei Z, Bähre H, König M (2021). cGAS-like receptors sense RNA and control 3′ 2′-cGAMP signalling in Drosophila. Nature.

[CR192] Sliter DA, Martinez J, Hao L, Chen X, Sun N, Fischer TD, Burman JL, Li Y, Zhang Z, Narendra DP (2018). Parkin and PINK1 mitigate STING-induced inflammation. Nature.

[CR193] Sotelo J, Corona T (2011). Varicella zoster virus and relapsing remitting multiple sclerosis. Mult Scler Int.

[CR194] Stetson DB, Medzhitov R (2006). Recognition of cytosolic DNA activates an IRF3-dependent innate immune response. Immunity.

[CR195] Stratmann SA, Morrone SR, van Oijen AM, Sohn J (2015). The innate immune sensor IFI16 recognizes foreign DNA in the nucleus by scanning along the duplex. Elife.

[CR196] Subramanian N, Natarajan K, Clatworthy MR, Wang Z, Germain RN (2013). The adaptor MAVS promotes NLRP3 mitochondrial localization and inflammasome activation. Cell.

[CR197] Sun L, Wu J, Du F, Chen X, Chen ZJ (2013). Cyclic GMP-AMP synthase is a cytosolic DNA sensor that activates the type I interferon pathway. Science.

[CR198] Taft J, Markson M, Legarda D, Patel R, Chan M, Malle L, Richardson A, Gruber C, Martín-Fernández M, Mancini GM (2021). Human TBK1 deficiency leads to autoinflammation driven by TNF-induced cell death. Cell.

[CR199] Tafuri F, Ronchi D, Magri F, Comi GP, Corti S (2015). SOD1 misplacing and mitochondrial dysfunction in amyotrophic lateral sclerosis pathogenesis. Front Cell Neurosci.

[CR200] Takaoka A, Wang Z, Choi MK, Yanai H, Negishi H, Ban T, Lu Y, Miyagishi M, Kodama T, Honda K (2007). DAI (DLM-1/ZBP1) is a cytosolic DNA sensor and an activator of innate immune response. Nature.

[CR201] Tan HY, Yong YK, Xue YC, Liu H, Furihata T, Shankar EM, Ng CS (2022). cGAS and DDX41-STING mediated intrinsic immunity spreads intercellularly to promote neuroinflammation in SOD1 ALS model. Iscience.

[CR202] Tanaka Y, Chen ZJ (2012). STING specifies IRF3 phosphorylation by TBK1 in the cytosolic DNA signaling pathway. Sci Signal.

[CR203] Tao J, Zhang X-W, Jin J, Du X-X, Lian T, Yang J, Zhou X, Jiang Z, Su X-D (2017). Nonspecific DNA binding of cGAS N terminus promotes cGAS activation. J Immunol.

[CR204] Thadathil N, Delotterie DF, Xiao J, Hori R, McDonald MP, Khan MM (2021). DNA double-strand break accumulation in Alzheimer’s disease: evidence from experimental models and postmortem human brains. Mol Neurobiol.

[CR205] Thapa RJ, Ingram JP, Ragan KB, Nogusa S, Boyd DF, Benitez AA, Sridharan H, Kosoff R, Shubina M, Landsteiner VJ (2016). DAI senses influenza A virus genomic RNA and activates RIPK3-dependent cell death. Cell Host Microbe.

[CR206] Udeochu JC, Amin S, Huang Y, Fan L, Torres ERS, Carling GK, Liu B, McGurran H, Coronas-Samano G, Kauwe G et al (2023) Tau activation of microglial cGAS–IFN reduces MEF2C-mediated cognitive resilience. Nat Neurosci 26(5):737–750 10.1038/s41593-023-01315-6 PMC10166855 37095396

[CR207] Unterholzner L, Keating SE, Baran M, Horan KA, Jensen SB, Sharma S, Sirois CM, Jin T, Latz E, Xiao TS (2010). IFI16 is an innate immune sensor for intracellular DNA. Nat Immunol.

[CR208] Varhaug KN, Vedeler CA, Myhr K-M, Aarseth JH, Tzoulis C, Bindoff LA (2017). Increased levels of cell-free mitochondrial DNA in the cerebrospinal fluid of patients with multiple sclerosis. Mitochondrion.

[CR209] Vila IK, Chamma H, Steer A, Saccas M, Taffoni C, Turtoi E, Reinert LS, Hussain S, Marines J, Jin L (2022). STING orchestrates the crosstalk between polyunsaturated fatty acid metabolism and inflammatory responses. Cell Metab.

[CR210] Vincent J, Adura C, Gao P, Luz A, Lama L, Asano Y, Okamoto R, Imaeda T, Aida J, Rothamel K (2017). Small molecule inhibition of cGAS reduces interferon expression in primary macrophages from autoimmune mice. Nat Commun.

[CR211] Wallings RL, Herrick MK, Tansey MG (2020). Parkinson’s disease: linking mitochondria to the immune response. Elife.

[CR212] Wang D, Gao H, Qin Q, Li J, Zhao J, Qu Y, Li J, Xiong Y, Min Z, Mao Z (2024) MicroRNA-218-5p-Ddx41 axis restrains microglia-mediated neuroinflammation through downregulating type I interferon response in a mouse model of Parkinson’s disease. J Transl Med 22(1):63. 10.1186/s12967-024-04881-w PMC10792813 38229084

[CR213] Wang Q, Westra J, van der Geest KS, Moser J, Bijzet J, Kuiper T, Lorencetti PG, Joosten LA, Netea MG, Heeringa P (2016). Reduced levels of cytosolic DNA sensor AIM2 are associated with impaired cytokine responses in healthy elderly. Exp Gerontol.

[CR214] Wang S-N, Guo X-Y, Tang J, Ding S-Q, Shen L, Wang R, Ma S-F, Hu J-G, Lü H-Z (2019). Expression and localization of absent in melanoma 2 in the injured spinal cord. Neural Regen Res.

[CR215] Wang Y, Fu Z, Li X, Liang Y, Pei S, Hao S, Zhu Q, Yu T, Pei Y, Yuan J (2021). Cytoplasmic DNA sensing by KU complex in aged CD4+ T cell potentiates T cell activation and aging-related autoimmune inflammation. Immunity.

[CR216] Wang Z, Choi MK, Ban T, Yanai H, Negishi H, Lu Y, Tamura T, Takaoka A, Nishikura K, Taniguchi T (2008). Regulation of innate immune responses by DAI (DLM-1/ZBP1) and other DNA-sensing molecules. Proc Natl Acad Sci USA.

[CR217] Watson RO, Manzanillo PS, Cox JS (2012). Extracellular M. tuberculosis DNA targets bacteria for autophagy by activating the host DNA-sensing pathway. Cell.

[CR218] Welch GM, Boix CA, Schmauch E, Davila-Velderrain J, Victor MB, Dileep V, Bozzelli PL, Su Q, Cheng JD, Lee A (2022). Neurons burdened by DNA double-strand breaks incite microglia activation through antiviral-like signaling in neurodegeneration. Sci Adv.

[CR219] Williamson RA, Burgoon MP, Owens GP, Ghausi O, Leclerc E, Firme L, Carlson S, Corboy J, Parren PW, Sanna PP (2001). Anti-DNA antibodies are a major component of the intrathecal B cell response in multiple sclerosis. Proc Natl Acad Sci USA.

[CR220] Wilson JE, Petrucelli AS, Chen L, Koblansky AA, Truax AD, Oyama Y, Rogers AB, Brickey W, Wang Y, Schneider M (2015). Inflammasome-independent role of AIM2 in suppressing colon tumorigenesis via DNA-PK and Akt. Nat Med.

[CR221] Wu P-J, Hung Y-F, Liu H-Y, Hsueh Y-P (2017). Deletion of the inflammasome sensor Aim2 mitigates Aβ deposition and microglial activation but increases inflammatory cytokine expression in an Alzheimer disease mouse model. Neuroimmunomodulation.

[CR222] Wu P-J, Liu H-Y, Huang T-N, Hsueh Y-P (2016). AIM 2 inflammasomes regulate neuronal morphology and influence anxiety and memory in mice. Sci Rep.

[CR223] Xie W, Lama L, Adura C, Tomita D, Glickman JF, Tuschl T, Patel DJ (2019). Human cGAS catalytic domain has an additional DNA-binding interface that enhances enzymatic activity and liquid-phase condensation. Proc Natl Acad Sci USA.

[CR224] Xie X, Ma G, Li X, Zhao J, Zhao Z, Zeng J (2023) Activation of innate immune cGAS-STING pathway contributes to Alzheimer’s pathogenesis in 5× FAD mice. Nat Aging 3(2):202–212 10.1038/s43587-022-00337-2 37118112

[CR225] Yanai H, Chiba S, Hangai S, Kometani K, Inoue A, Kimura Y, Abe T, Kiyonari H, Nishio J, Taguchi-Atarashi N (2018). Revisiting the role of IRF3 in inflammation and immunity by conditional and specifically targeted gene ablation in mice. Proc Natl Acad Sci USA.

[CR226] Yang H, Wang H, Ren J, Chen Q, Chen ZJ (2017). cGAS is essential for cellular senescence. Proc Natl Acad Sci USA.

[CR227] Yoh SM, Schneider M, Seifried J, Soonthornvacharin S, Akleh RE, Olivieri KC, De Jesus PD, Ruan C, de Castro E, Ruiz PA (2015). PQBP1 is a proximal sensor of the cGAS-dependent innate response to HIV-1. Cell.

[CR228] Yu CH, Davidson S, Harapas CR, Hilton JB, Mlodzianoski MJ, Laohamonthonkul P, Louis C, Low RRJ, Moecking J, De Nardo D (2020). TDP-43 triggers mitochondrial DNA release via mPTP to activate cGAS/STING in ALS. Cell.

[CR229] Yu H, Liao K, Hu Y, Lv D, Luo M, Liu Q, Huang L, Luo S (2022). Role of the cGAS-STING pathway in aging-related endothelial dysfunction. Aging Dis.

[CR230] Yu J, Zhou X, Chang M, Nakaya M, Chang J-H, Xiao Y, William Lindsey J, Dorta-Estremera S, Cao W, Zal A (2015). Regulation of T-cell activation and migration by the kinase TBK1 during neuroinflammation. Nat Commun.

[CR231] Yum S, Li M, Fang Y, Chen ZJ (2021). TBK1 recruitment to STING activates both IRF3 and NF-κB that mediate immune defense against tumors and viral infections. Proc Natl Acad Sci USA.

[CR232] Zhang C, Shang G, Gui X, Zhang X, Bai X-c, Chen ZJ (2019). Structural basis of STING binding with and phosphorylation by TBK1. Nature.

[CR233] Zhang J, Perry G, Smith MA, Robertson D, Olson SJ, Graham DG, Montine TJ (1999). Parkinson’s disease is associated with oxidative damage to cytoplasmic DNA and RNA in substantia nigra neurons. Am J Pathol.

[CR234] Zhang T, Yin C, Boyd DF, Quarato G, Ingram JP, Shubina M, Ragan KB, Ishizuka T, Crawford JC, Tummers B (2020). Influenza virus Z-RNAs induce ZBP1-mediated necroptosis. Cell.

[CR235] Zhang X, Wu J, Du F, Xu H, Sun L, Chen Z, Brautigam CA, Zhang X, Chen ZJ (2014). The cytosolic DNA sensor cGAS forms an oligomeric complex with DNA and undergoes switch-like conformational changes in the activation loop. Cell Rep.

[CR236] Zhang Z, Bao M, Lu N, Weng L, Yuan B, Liu Y-J (2013). The E3 ubiquitin ligase TRIM21 negatively regulates the innate immune response to intracellular double-stranded DNA. Nat Immunol.

[CR237] Zhang Z, Yuan B, Bao M, Lu N, Kim T, Liu Y-J (2011). The helicase DDX41 senses intracellular DNA mediated by the adaptor STING in dendritic cells. Nat Immunol.

[CR238] Zhao Y, Simon M, Seluanov A, Gorbunova V (2023). DNA damage and repair in age-related inflammation. Nat Rev Immunol.

[CR239] Zheng W, Zhou R, Li S, He S, Luo J, Zhu M, Chen N, Chen H, Meurens F, Zhu J (2020). Porcine IFI16 negatively regulates cGAS signaling through the restriction of DNA binding and stimulation. Front Immunol.

[CR241] Zhou W, Whiteley AT, de Oliveira Mann CC, Morehouse BR, Nowak RP, Fischer ES, Gray NS, Mekalanos JJ, Kranzusch PJ (2018). Structure of the human cGAS–DNA complex reveals enhanced control of immune surveillance. Cell.

[CR242] Zhou Z, Zhang X, Lei X, Xiao X, Jiao T, Ma R, Dong X, Jiang Q, Wang W, Shi Y (2021). Sensing of cytoplasmic chromatin by cGAS activates innate immune response in SARS-CoV-2 infection. Signal Transduct Target Ther.

